# Synthesis of Os Hydride Complexes Supported by the Diarylamido/Bis(phosphine) PNP Ligand and Attempts at Using (PNP)Ru and (PNP)Os Complexes in C–H Borylation Catalysis

**DOI:** 10.1021/acs.organomet.4c00388

**Published:** 2024-11-12

**Authors:** Patricio Castillo, Bryan J. Foley, Samuel R. Lee, Billy J. McCulloch, Nattamai Bhuvanesh, Oleg V. Ozerov

**Affiliations:** Department of Chemistry, Texas A&M University, College Station, Texas 77842, United States

## Abstract

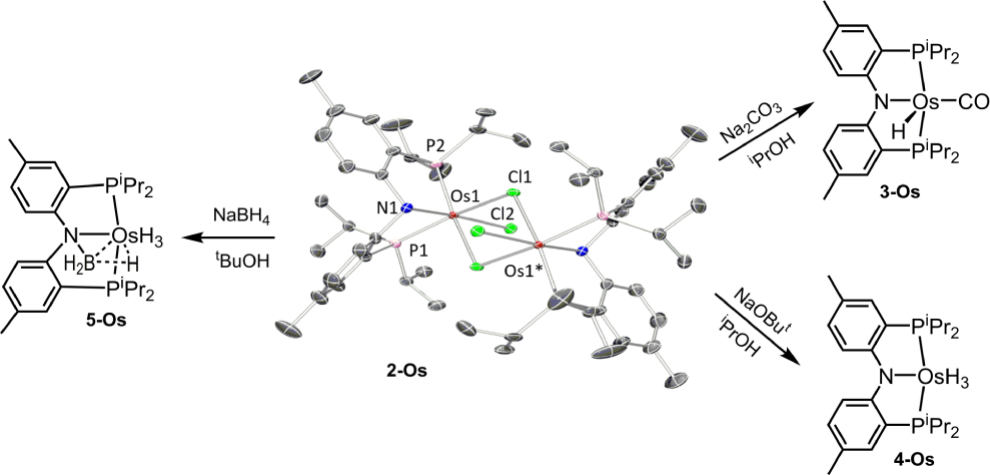

This manuscript describes the synthesis of Os complexes supported by the diarylamido/bis(phosphine) PNP pincer ligand. Compound (PNP)OsH(CO) (**3-Os**) was prepared by analogy with the previously reported **3-Ru**. However, attempts to make (PNP)OsH_3_ (**4-Os**) analogously to **4-Ru** resulted in the formation of an unexpected compound (**5-Os**) that is a product of addition of a BH_3_ unit across the Os–N bond in **4-Os**. Nonetheless, **4-Os** was prepared via an alternative route. Unlike **4-Ru**, **4-Os** appears to be a classical trihydride. Compounds **3-Ru**, **3-Os**, **4-Os**, **4-Ru**, and **5-Os** were tested as potential catalysts for (a) dehydrogenative borylation of terminal alkynes (DHBTA) and (b) dehydrogenative borylation of benzene. No catalytic C–H borylation was observed for any of them, but all of them catalyzed unselective hydroboration of 4-MeC_6_H_4_CCH.

## Introduction

**Figure 1:**
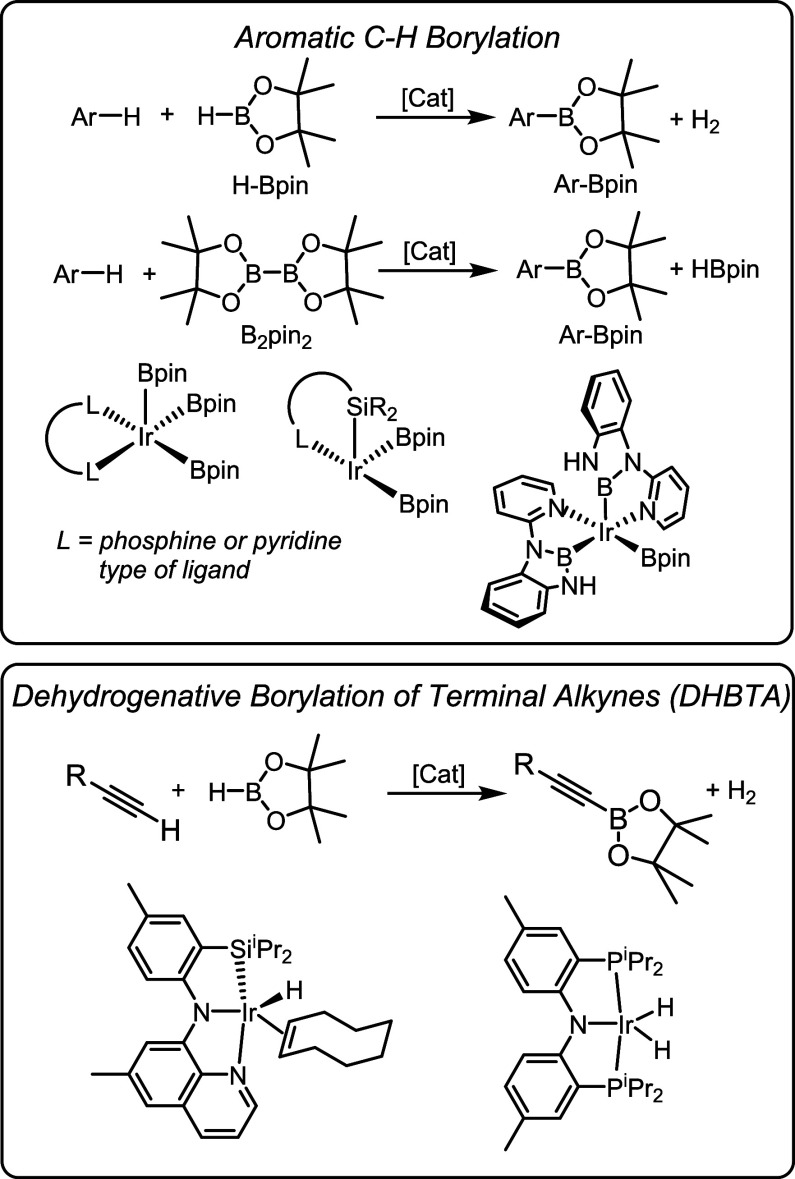
Top: aromatic C–H borylation and selected Ir catalysts. Bottom: DHBTA and previously reported pincer-supported Ir catalysts.

**Scheme 1:**
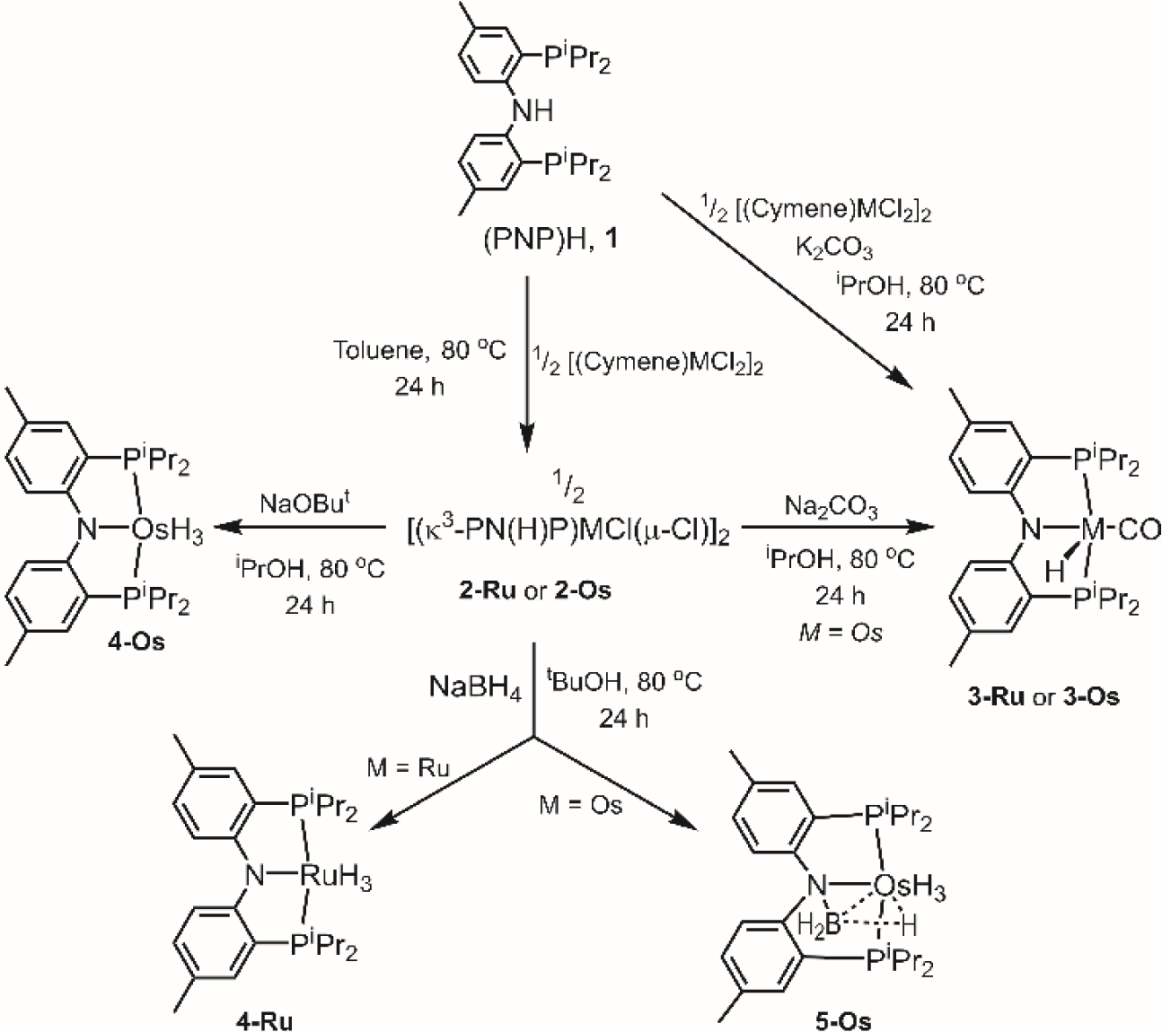
Synthesis of the Previously Reported (PNP)Ru and New (PNP)Os Compounds

Catalytic borylation of C–H bonds is a widely studied reaction^1,2^ whose synthetic value is in the efficient production of organoboronates,^3^ versatile building blocks in synthesis. Among the transition metals, complexes of Ir have been among the first and among the most highly active catalysts, especially as pertains to the aromatic C–H borylation.^4−7^ Over the past decade, our group has explored dehydrogenative borylation of terminal alkynes (DHBTA, Figure 1)^8−12^ to chemoselectively produce alkynylboronates.^13,14^ We were able to develop highly effective catalysts based on Ir complexes supported by diarylamido-centered pincer ligands. These Ir DHBTA catalysts are inactive in aromatic C–H borylation, while the common Ir catalysts for aromatic C–H borylation are poisoned by alkynes. DHBTA catalysts utilizing Zn,^15−20^ Co,^21^ Fe,^22^ Cu,^23,24^ Pd,^25^ Mg,^26^ Al,^27^ Mn,^28^ and phosphorus superbases^18^ have been reported in the literature, as well as select boron reagents for dehydrogenative stoichiometric reactions.^29^ Clearly, the DHBTA reactivity is possible with catalysts based on a great variety of elements. We thus became interested in whether complexes of some other precious metals might display C–H borylation activity when supported by ligands that we employed with Ir. We previously established that Rh complexes were not active,^30^ and turned our attention to Ru and Os. Some Ru complexes have been previously used for C–H borylation catalysis but not in the context of DHBTA or in a pincer framework.^31−34^ C–H activation reactivity of pincer complexes of Os^35−37^ and the chemistry of boryl-Os compounds^38−40^ have been examined, but it does not appear that Os compounds have been used for C–H borylation catalysis. We previously reported Ru complexes (PNP)RuH(CO) (**3-Ru**) and (PNP)RuH_3_ (**4-Ru**), which were prepared from the diarylamine/bis(phosphine) (PNP)H^41^ ligand **1** via the intermediacy of **2-Ru** (Scheme 1).^42^ In this study, we disclose the syntheses of their Os analogs, as well as an unexpected new boron-containing Os polyhydride complex. Although the screening of these compounds for potential C–H borylation activity was not fruitful, the study brings forth synthetic and structural insight.

## Results and Discussion

### Synthesis of Os Complexes

The preparation of the Os analogs [(PN(H)P)OsCl_2_]_2_ (**2-Os**) and the (PNP)OsH(CO) (**3-Os**) proceeded very similarly to Ru (Scheme 1).^42^ Thermolysis of the (PNP)H ligand (**1**) with [(cymene)OsCl_2_]_2_ at 80 °C overnight resulted in the formation of **2-Os**. It was only obtained in ca. 95% purity, but that was adequate for the use in further syntheses. (PNP)OsH(CO) (**3-Os**) was prepared in 40% isolated yield from **2-Os** by subjecting it to thermolysis in isopropanol in the presence of excess Na_2_CO_3_. In the synthesis of **3-Ru**, we previously demonstrated that the carbonyl ligand can be derived from CO_2_.^42^ We assume that a similar process takes place with Os, but we have not investigated this matter in detail.

On the other hand, the attempted synthesis of **4-Os** by a calque from the Ru procedure (**2-Os** + NaBH_4_ in ^*t*^BuOH) unexpectedly resulted in the formation of compound **5-Os**, isolated in 86% yield. It can be formulaically regarded as the product of addition of BH_3_ to **4-Os**. To circumvent the formation of **5-Os**, **2-Os** was treated with NaOBu^*t*^ in isopropanol. In this case, isopropoxide formed in situ served as the hydride donor and isopropanol served as the source of extra H_2_. This procedure resulted in the isolation of **4-Os** in 40% yield after workup.

### Spectroscopic Characterization

Compound **3-Os** gave rise to a single hydride resonance in its ^1^H NMR spectrum at δ −30.68 ppm, displaying the expected coupling to two^31^P nuclei (^2^*J*_H–P_ = 13 Hz). This chemical shift value is close to the electronically similar five-coordinate Os complexes (P^*i*^Pr_3_)_2_OsHClCO (δ −31.9 ppm)^43^ and (^Si^PNP)OsH(CO)^44^ (δ −29.4 ppm, ^Si^PNP is a disilylamido/bis(phosphine) pincer), corresponding to a hydride *trans* to an empty site in a geometry close to square-pyramidal.^45^

Compound **4-Os** displayed a single hydride resonance of intensity 3H at δ −16.03 ppm (broad singlet). This chemical shift value is similar to that noted for (^Si^PNP)OsH_3_ (−16.53 ppm), which was reported as a classical trihydride.^44^ Dissolution of **4-Os** in C_6_D_6_ at ambient temperature led to near-complete H/D exchange of the Os–H positions with C–D within 20 min, resulting in the observation of only a single isotopomer by ^1^H NMR spectroscopy, presumably (PNP)OsD_2_H (**4-Os-***d*_2_). Addition of C_6_H_5_F to such a solution led to the emergence of the two other isotopomers ((PNP)OsH_3_ (**4-Os**) and (PNP)OsH_2_D (**4-Os-***d*_1_) over time. Each of the isotopomeric hydride resonances presented as a broadened resonance with a hint of the triplet substructure owing to ^1^H–^31^P coupling. Explicit H–D coupling was not perceptible in these resonances and the shapes and line widths of the resonances from the three different isotopomers were not significantly different. This suggests that the magnitude of the H–D coupling in the isotopomers of **4-Os** is small (probably not exceeding 1–2 Hz). This is consistent with a trihydride formulation; if **4-Os** contained a dihydrogen ligand, the apparent *J*_H–D_ value would be much higher (even if averaged in the H/H_2_ system).^46−50^

At ambient temperature, **5-Os** presented a broad signal of intensity 2H at δ 2.70 ppm assigned to the two BH hydrogens and a broad resonance of intensity 4H (δ −10.0 ppm) assigned to the four Os-bound hydrogens. Observing **5-Os** in toluene-*d*_8_ while lowering the temperature revealed that the apparent 4H resonance at RT splits into three resonances of relative intensity 1:1:2 (δ −8.7, −10.4, and −11.3 ppm at −90 °C). The middle resonance showed a discernible triplet substructure between −20 and −70 °C. From the shape of the resonances at various temperatures, it appears that the outer two coalesce at around −20 °C without much change in the central (triplet) resonance. It is not clear whether another process causes coalescence of the central triplet resonance with the rest at 25 °C, or if the appearance of coalescence is due to chemical shift coincidence. We tentatively assign the middle (triplet) resonance to the Os–H *trans* to B–H, the broad resonance of intensity 1H to the B–H–Os hydrogen and the resonance of intensity 2H to the remaining two hydrides, ostensibly exchanging with B–H–Os. The BH_2_ resonance remained relatively unperturbed in the temperature range of the study, indicating that it does not exchange with the OsH_4_ hydrogens at a rate that would influence NMR spectra.

### XRD Structural Characterization

**Figure 2:**
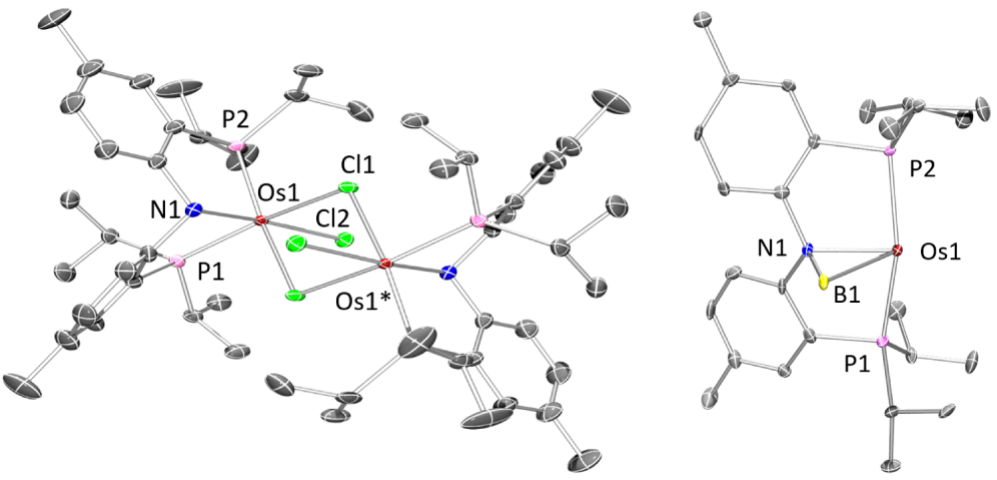
ORTEP drawings (50% probability ellipsoids) of **2-Os** (left) and **5-Os** (right). Only one of the two independent molecules of **5-Os** is shown. Hydrogen atoms and the disordered pentane molecule in the structure of **5-Os** are omitted for clarity. Selected angles (deg) and distances (Å) for **2-Os** follow: Os1–N1, 2.128(2); Os1–P1, 2.2682(11); Os1–P2, 2.2275(10); Os1–Cl1, 2.4543(12); Os1–Cl1*, 2.4885(9); Os1–Cl2, 2.4294(9); P1–Os1–P2, 100.04(3); Cl1–Os1–Cl1*, 77.72(2). Selected angles (deg) and distances (Å) for **5-Os**, **molecule 1**, follow: Os1–N1, 2.155(5); Os1–B1, 2.421(7); Os1–P1, 2.3000(18); Os1–P2, 2.2847(18); N1–B1, 1.568(9); Os1–B1–N1, 61.0(3); P1–Os1–P2, 160.85(6). Selected angles (deg) and distances (Å) for **5-Os**, **molecule 2**, follow: Os1–N1, 2.176(5); Os1–B1, 2.415(8); Os1–P1, 2.3046(17); Os1–P2, 2.2948(18); N1–B1, 1.571(10); Os1–B1–N1, 62.0(3); P1–Os1–P2, 161.97(7).

The structures of **2-Os** and **5-Os** were determined via X-ray diffraction studies on suitable single crystals (Figure 2). The [(PNHP)OsCl_2_]_2_ dimer (**2-Os**) lies on a center of symmetry in the crystal that relates the two (PNHP)OsCl_2_ fragments. The dimer is formed by means of a pair of bridging chlorides, resulting in an approximately octahedral environment about Os. The tridentate ligand binds to Os facially. The presence of the amine (NH) moiety is inferred from the pyramidalized geometry at N, which in turn permits close approach of the P–Os–P angle (ca. 100°) to the idealized 90°.

The structure solution of **5-Os** revealed two independent molecules in the asymmetric unit; they possess very similar molecular geometries. The hydrogen atom positions were not reliably obtained in **5-Os**. The boron atom is bridging N and Os. The Os–B distance of ca. 2.42 Å is much longer than the Os–B distance in σ-BH complexes of catechol- or pinacolborane or the Os–B distances in the related osmium-boryls (ca. 2.0–2.1 Å).^51^ It is closer to the Os–B distances recorded in (R_3_P)_2_OsH_3_(κ^2^-H_2_BH_2_) (2.30(1) Å)^52^ and (R_3_P)_2_OsH_3_(κ^2^-H_2_BR_2_) (2.355(3) Å; *R* = various alkyls),^53^ or in (Ph_3_P)_2_OsH(CO)(B_3_H_8_) (2.44–2.48 Å).^54^ The Os–B distance in **5-Os** is also comparable to some of the longer Ir–B distances observed in Ir **→** BR_3_ complexes.^55^

### Attempts at DHBTA and Aromatic C–H Borylation Catalysis

Complexes of type **3** and **4**, as well as **5-Os** were tested as potential catalysts for the DHBTA reaction between 4-MeC_6_H_4_CCH and HBpin (used in a 1:2 ratio, Table 1). The reactions were conducted using 1 mol% of the transition metal complex relative to 4-MeC_6_H_4_CCH. These mixtures were thermolyzed in C_6_D_6_ for 3 d at 80 °C and analyzed by NMR spectroscopy. The DHBTA product 4-MeC_6_H_4_CCBpin was not detected in any of the five cases. Instead, the products of hydroboration^56^ of 4-MeC_6_H_4_CCH were detected with the conversion ranging from 25% to 95%. The ratio of *trans*-CH_3_–C_6_H_4_–CH=CH-Bpin and *cis*-CH_3_–C_6_H_4_–CH=CH-Bpin in the reaction with **3-Ru** was ca. 20:1 and it varied between 2:1 and 1:1 for the other reactions. Examples of selective catalytic hydroboration of alkynes do exist,^57,58^ including by a (PNP*)RuH_4_ polydydride complex (PNP* = pyridine/bis(phosphine) pincer ligand).^59^

**Table 1:**
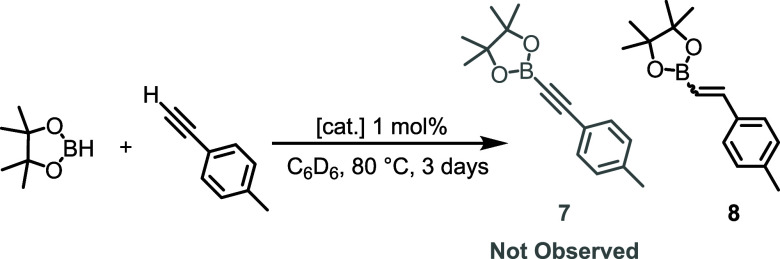
Attempts at Catalytic DHBTA of 4-Ethynyltoluene Using (PNP)Ru and Os Complexes

entry	[cat.]	% conv.	% products^a^
1	**3-Ru**	99	0/95/5
2	**4-Ru**	88	0/55/45
3	**3-Os**	25	0/72/28
4	**4-Os**	25	0/72/28
5	**5-Os**	35	0/51/49

a Product yields listed as 7/(*E*)-**8**/(*Z*)-**8**.

In addition to testing for DHBTA activity, we decided to evaluate the potential of compounds **3**–**5** in aromatic C–H (C–D) borylation. Experiments were conducted in C_6_D_6_ as solvent, with 5% of the transition metal complex relative to the 1:1 mixture of HBpin and 1-hexene. These conditions were modeled after our recent work on C–H borylation of arenes using (pincer)Ir catalysts.^60,61^ After 3 d at 80 °C, NMR analysis revealed only the formation of isomers of 1-hexene, with no evidence for any C–H borylation products.

## Conclusion

In summary, we prepared new Os hydride complexes supported by the diarylamido/bis(phosphine) PNP ligand. These compounds, along with the previously described Ru analogs were tested as potential catalysts of C–H borylation of sp and sp^2^ C–H bonds, but they did not show any C–H borylation activity. The complexes did show modest activity in hydroboration of a terminal alkyne, with little regioselectivity.

## Experimental Section

### General Considerations

Unless specified otherwise, all manipulations were performed under an Ar atmosphere using standard Schlenk line or glovebox techniques. Toluene, diethyl ether, pentane, benzene, C_6_D_6_ were dried over NaK/Ph_2_CO/18-crown-6, distilled or vacuum transferred and stored over molecular sieves in an Ar-filled glovebox. Ligand **1** was prepared according to the published procedure.^41^ The Ru complexes **2-Ru**, **3-Ru**, and **4-Ru** were prepared as described previously,^42^ but using [(Cymene)RuCl_2_]_2_ instead of [(COD)RuCl_2_]_*n*_. Alkynes were deoxygenated by three freeze–pump–thaw cycles prior to use. All other chemicals were used as received from commercial vendors. NMR spectra were recorded on a Varian Inova 300, Mercury 300 (^1^H NMR, 299.952 MHz; ^13^C NMR, 75.421 MHz), Varian Inova 400 (^1^H NMR, 399.535 MHz; ^11^B NMR, 128.185 MHz; ^13^C NMR, 100.465 MHz), and NMRS 500 (^1^H NMR, 499.703 MHz; ^13^C NMR, 125.697 MHz; ^31^P NMR, 202.183 MHz) spectrometer. Chemical shifts are reported in δ (ppm). For ^1^H and ^13^C NMR spectra, the residual solvent peak was used as an internal reference. ^31^P NMR spectra were referenced externally to δ = 0 ppm by using H_3_PO_4_. ^11^B NMR spectra were referenced externally to δ = 0 ppm by using BF_3_·Et_2_O. Elemental analyses were performed by CALI Laboratories, Inc. (Parsippany, NJ).

### [(PN(H)P)OsCl_2_]_*n*_ (**2-Os**)

In an Ar-filled glovebox, the following were added to a culture tube: **1** (0.559 g, 0.00130 mol), [(cymene)OsCl_2_]_n_ (0.400 g, 0.000653 mol) and 15 mL of freshly distilled and degassed toluene. The culture tube was then Teflon taped up and taken outside the box, where it then stirred at 80 °C overnight. The yellow-orange precipitate was collected by filtration, and dried under vacuum. Yield: 0.49 g (52%). ^1^H NMR (CD_2_Cl_2_, 500 MHz): δ 9.76 (s, 2H, PN(*H*)P), 7.90 (m, 4H, Ar-*H*), 7.19 (m, 4H, Ar-*H*), 7.07 (m, 4H, Ar-*H*), 2.83 (m, 4H, C*H*Me_2_), 2.31 (s, 12H, Ar-*Me*), 2.03 (m, 4H, C*H*Me_2_), 1.29 (dvt, 24 H, CH*Me*_2_), 0.89 (m, 12H, CH*Me*_2_), 0.60 (m, 12H, CH*Me*_2_).

### (PNP)OsH(CO) (**3-Os**)

In an Ar-filled glovebox, **2-Os** (310 mg, 0.224 mmol) and Na_2_CO_3_ (137 mg, 1.43 mmol) were measured out into a 25 mL Schlenk flask, with 10 mL of isopropanol as solvent. The flask was taken out of the glovebox where it stirred and heated in an oil bath at 80 °C overnight. The solvent in the flask was removed *in vacuo* on the Schlenk line outside the box, and was then taken back inside the box where the residual solid was extracted with toluene through a filter pipet into another Schlenk flask. The solvent was then evaporated to dryness, affording a dark red solid as the final product. The solid was recrystallized in pentane. Yield: 113 mg (40%). ^1^H NMR (C_6_D_6_, 400 MHz): δ 7.70 (d, 2H, *J* = 8.4 Hz, Ar-*H*), 6.95 (s, 2H, Ar-*H*), 6.82 (d, 2H, *J* = 8.4 Hz, Ar-*H*), 2.49 (m, 2H, C*H*Me_2_), 2.17 (s, 6H, Ar-*Me*), 2.11 (m, 2H, C*H*Me_2_), 1.29–1.18 (m, 12H, CH*Me*_*2*_), 1.00 (dvt, 6H, CH*Me*_*2*_), 0.94 (dvt, 6H, CH*Me*_*2*_), −30.68 (t, 1H, *J =* 12.7 Hz, Os-*H*). ^31^P{^1^H} NMR (C_6_D_6_, 162 MHz): δ 60.7. ^13^C{^1^H} NMR (C_6_D_6_, 101 MHz): δ 191.8 (t, *J*_C–P_ = 9.5 Hz, Os-*C*O), 164.6 (t, *J*_C–P_ = 12.6 Hz, Ar-*C*-P), 132.8, 131.7, 127.5 (t, *J*_C–P_ = 3.1 Hz, *C*H(CH_3_)_2_), 125.1 (t, *J*_C–P_ = 17.1 Hz, *C*H(CH_3_)_2_), 116.2 (t, *J*_C–P_ = 5.7 Hz, *Ar*C), 27.6 (t, *J*_C–P_ = 11 Hz, *C*HMe_2_), 25.5 (t, *J*_C–P_ = 13.4 Hz, *C*HMe_2_), 20.4 (s, Ar-*Me*), 19.5 (t, *J*_C–P_ = 3 Hz, CH*Me*_2_), 19.3 (t, *J*_C–P_ = 3 Hz, CH*Me*_2_), 18.6 (s, CH*Me*_22_) 18.2 (s, CH*Me*_2_). Elem. anal. calcd for C_27_H_41_NOOsP_2_: C, 50.06; H, 6.38; N, 2.16. Found: C, 49.59; H, 6.63; N, 2.13.

### (PNP)OsH_3_ (**4-Os**)

In an Ar-filled glovebox, **2-Os** (310 mg, 0.224 mmol) and sodium *tert*-butoxide (137 mg, 1.43 mmol) were measured out into a 25 mL Schlenk flask, with 10 mL of isopropanol as solvent. The flask was taken out of the glovebox where it stirred and heated in an oil bath at 80 °C overnight. The solvent in the flask was removed *in vacuo* on the Schlenk line outside the box, and was then taken back inside the box where the residual solid was extracted with toluene through a filter pipet into another Schlenk flask. The solvent was then evaporated to dryness, affording a dark red solid as the final product. The solid was recrystallized in pentane. Yield: 113 mg (40%). ^1^H NMR (C_6_D_6_, 400 MHz): δ 7.82 (dt, *J* = 8.4, 2.0 Hz, Ar-*H*), 7.01 (d, *J* = 5.2 Hz, 2H, Ar-*H*), 6.87 (dd, *J* = 8.5, 2.1 Hz, Ar-*H*), 2.20 (s, 6H, Ar-*Me*), 2.06 (m, 4H, C*H*Me_2_), 1.16 (m, 12H, CH*Me*_2_), 0.93 (dvt, 6H, *J*_*HH*_ = *J*_*HP*_ = 7.0 Hz, C*H*Me_2_), −16.04 (s, 3H, Os-*H*). ^31^P{^1^H} NMR (C_6_D_6_, 121 MHz): δ 57.9. ^13^C{^1^H} NMR (C_6_D_6_, 101 MHz): δ 166.1 (s, Ar*C*), 137.9 (s, Ar*C*) 133.1 (s, Ar*C*), 131.0 (s, Ar*C*), 129.2 (t, *J*_C–P_*=* 1.3 Hz, Ar*C*), 115.6 (t, *J*_C–P_ = 5.1 Hz, Ar*C*), 26.0 (overlapping signals, *C*HMe_2_), 20.4 (s, Ar-*Me*), 20.0 (t, *J*_C–P_ = 3.5 Hz, CH*Me*_2_), 18.6 (s, CH*Me*_2_). Elem. anal. calcd for C_26_H_43_NOsP_2_: C, 50.22; H, 6.97; N, 2.25. Found: C, 50.22; H, 6.22; N, 2.08.

### Hydrogen–Deuterium Exchange in **4-Os**

To a J. Young tube, 15 mg of **4-Os** (0.025 mmol) was loaded with 0.4 mL C_6_D_6_ at 21.0 °C. An intense residual solvent peak and weak, broad singlet in the hydride region of the ^1^H NMR spectrum (taken 20 min after sample preparation) indicated significant H/D exchange between **4-Os** and the solvent. To the solution, 0.20 mL of fluorobenzene (1.09 mmol) was added, and the J. Young tube shaken, the emergence and changing populations of **4-Os**, **4-Os-***d*_1_, and **4-Os-***d*_*2*_ were monitored by ^1^H NMR spectroscopy.

### (PNP)(BH_2_)OsH_4_ (**5-Os**)

In an Ar-filled glovebox, a culture tube was filled with **2-Os** (260 mg, 0.188 mmol), NaBH_4_ (164 mg, 4.33 mmol), and 10 mL *tert*-butanol before the tube was placed in an oil bath at 80 °C with stirring overnight. Volatiles were removed under vacuum, and the residue was suspended in pentane and filtered through a plug of Celite. Solvent was then removed under vacuum, affording a light brown solid. The solid was recrystallized in pentane. Yield: 200 mg (86%). ^1^H NMR (C_6_D_6_, 500 MHz, 298.15 K): δ 7.75 (d, 2H, *J* = 8.4 Hz, Ar-*H*), 7.04 (s, 2H, Ar-*H*), 6.72 (d, 2H, 8.4 Hz, Ar-*H*), 2.68 (brs, 2H, B-*H*), 2.22 (m, 2H, C*H*Me_2_), 2.11 (s, 6H, Ar–C*H*_3_), 1.86 (m, 2H, C*H*Me_2_), 1.24 (dvt, 6H, CH*Me*_*2*_, *J*_*HH*_ = *J*_*HP*_ = 7.0 Hz), 1.16 (dvt, 6H, CH*Me*_2_, *J*_*HH*_ = *J*_*HP*_ = 6.7 Hz), 1.09 (dvt, 6H, CH*Me*_*2*_, *J*_*HH*_ = *J*_*HP*_ = 7.4 Hz), 0.82 (dvt, 6H, CH*Me*_*2*_, *J*_*HH*_ = *J*_*HP*_ = 6.9 Hz), −10.0 (brs, 4H, Os-*H*). ^1^H NMR (C_6_D_6_, 500 MHz, 183.15 K, Hydride region): δ −8.6 (brs, Os–*H*, 1H), −10.4 (brs, Os–*H*, 1H), −11.2 (brs, Os–*H*, 2H). ^31^P{^1^H} NMR (C_6_D_6_, 202 MHz): δ 51.6. ^11^B NMR (C_6_D_6_, 128 MHz): δ −11.8. ^13^C{^1^H} NMR (C_6_D_6_, 120 MHz): δ 162.2 (t, *J*_C–P_ = 8.3 Hz, Ar*C*), 135.9 (t, *J*_C–P_ = 16.4 Hz, Ar*C*), 133.7 (t, *J*_C–P_ = 2.6 Hz, Ar*C*), 132.7 (s, Ar*C*), 129.2 (s, Ar*C*), 123.0 (t, *J*_C–P_ = 4.4 Hz, Ar*C*), 27.9 (t, *J*_C–P_ = 12.3 Hz, *C*HMe_2_), 26.1 (t, *J*_C–P_ = 16.7 Hz, *C*HMe_2_), 22.2 (t, *J*_C–P_ = 2.9 Hz, Ar-*Me*), 20.9 (t, *J*_C–P_ = 4.3 Hz, CH*Me*_*2*_), 20.4 (s, CH*Me*_*2*_), 20.1 (s, CH*Me*_*2*_), 19.9 (t, *J*_C–P_ = 2.2 Hz, CH*Me*_2_). Elem. anal. calcd for C_26_H_46_BNOsP_2_ × (C_5_H_12_)_0.5_: C, 50.96; H, 7.80. Found: C, 50.28; H, 7.61. The slight discrepancy in the elemental analysis results is likely owing to the less than stoichiometric amount of pentane (a disordered component of the X-ray structure solution at 0.5 equiv. per Os) in the solid.

### General Procedure for Attempted Catalysis of DHBTA

To a J. Young NMR tube in an Ar-filled glovebox, 35 μL (1.0 μmol, 0.01 M in C_6_D_6_) of catalyst (**3-Ru**, **4-Ru**, **3-Os**, **4-Os**, and **5-Os**) and 50 μL HBpin (0.20 mmol) were added sequentially via microsyringe. The tube was shaken to allow the contents to evenly mix throughout. After this, 4-ethynyltoluene (35 μL, 0.10 mmol) was dissolved in 380 μL C_6_D_6_. This solution was added to the J. Young tube in four parts in 1 min intervals. This mixture was heated in an oil bath at 80 °C for 3 days. ^1^H NMR features of **(E)-8**(62) and **(Z)-8**(63) were in agreement with those in the literature, and are reported herein. **(E)-8:**^1^H NMR (500 MHz, C_6_D_6_): δ 7.40 (d, ^3^J_H–H_ = 8.0 Hz, 2H, Ar-*H*), 7.38 (d, ^3^J_H–H_ = 19 Hz, 1H, alkenyl-*H*), 7.15 (d, ^3^J_H–H_ = 8.0 Hz, 2H, Ar-*H*), 6.12 (d, ^3^J_H–H_ = 19 Hz, 1H, alkenyl-*H*), 2.35 (s, 3H, Ar-*Me*), 1.32 (s, 12H, *Me* on Bpin). **(Z)-8:**^1^H NMR (500 MHz, C_6_D_6_): δ 7.47 (d, ^3^J_H–H_ = 8.0 Hz, 2H, Ar-*H*), 7.19 (d, ^3^J_H–H_ = 15 Hz, 1H, alkenyl-*H*), 7.12 (d, ^3^J_H–H_ = 8.0 Hz, 2H, Ar-*H*), 5.54 (d, ^3^J_H–H_ = 15 Hz, 1H, alkenyl-*H*), 2.36 (s, 3H, Ar-*Me*), 1.31 (s, 12H, *Me* on Bpin).

### Results of Attempted DHBTA Catalysis Using **3-Ru**

A 0.010 M stock solution of **3-Ru** was used in this case. General procedure stands. After 3 d of heating, analysis by ^1^H NMR spectroscopy revealed the reaction went to 99% completion, affording 95% *trans*-CH_3_–C_6_H_4_–CH=CH-Bpin and 5% *cis*-CH_3_–C_6_H_4_–CH = CH-Bpin.

### Results of Attempted DHBTA Catalysis Using **4-Ru**

A 0.01 M stock solution of **4-Ru** was used in this case. General procedure stands. After 3 d of heating, analysis by ^1^H NMR spectroscopy revealed the reaction went to 88% completion, affording 48% *trans*-CH_3_–C_6_H_4_–CH=CH-Bpin and 40% *cis*-CH_3_–C_6_H_4_–CH=CH-Bpin.

### Results of Attempted DHBTA Catalysis Using **3-Os**

A 0.010 M stock solution of **3-Os** was used in this case. General procedure stands. After 3 d of heating, analysis by ^1^H NMR spectroscopy revealed the reaction went to 25% completion, affording 18% *trans*-CH_3_–C_6_H_4_–CH=CH-Bpin and 7% *cis*-CH_3_–C_6_H_4_–CH=CH-Bpin.

### Results of Attempted DHBTA Catalysis Using **4-Os**

A 0.010 M stock solution of **4-Os** was used in this case. General procedure stands. After 3 d of heating, analysis by ^1^H NMR spectroscopy revealed the reaction went to 25% completion, affording 18% *trans*-CH_3_–C_6_H_4_–CH==CH-Bpin and 7% *cis*-CH_3_–C_6_H_4_–CH=CH-Bpin.

### Results of Attempted DHBTA Catalysis Using **5-Os**

A 0.010 M stock solution of **5-Os** was used in this case. General procedure stands. After 3 d of heating, analysis by ^1^H NMR spectroscopy revealed the reaction went to 35% completion, affording 18% *trans*-CH_3_–C_6_H_4_–CH=CH-Bpin and 17% *cis*-CH_3_–C_6_H_4_–CH=CH-Bpin.

### General Procedure for Attempted Arene Borylation

To a J. Young NMR tube, 35 μL (1.0 μmol, 0.01 M in C_6_D_6_) of catalyst (**3-Ru**, **4-Ru**, **3-Os**, **4-Os**, and **5-Os**), 50 μL of HBpin (0.08 mmol), and 45 μL 1-hexene (0.08 mmol), and 370 μL were added sequentially via microsyringe before the tube was placed in an 80 °C oil bath to heat for 3 days. For all catalysts, 1-hexene isomerization products were observed^64^ with no evidence of arene borylation.

## Supporting Information Available

Details of X-ray diffraction experiments and pictorial NMR spectra (PDF)
