# Supplementary material for: Synthesis of Os Hydride Complexes Supported by the Diarylamido/Bis(phosphine) PNP Ligand and Attempts at Using (PNP)Ru and (PNP)Os Complexes in C–H Borylation Catalysis
Source: Organometallics. 2024 Nov 12;43(22):2944–50. doi: 10.1021/acs.organomet.4c00388 (PMC11600505; doi:10.1021/acs.organomet.4c00388)
Supplement: Supplementary file 1 — om4c00388_si_001.pdf [file om4c00388_si_001.pdf]

Supporting Information

Synthesis of Os Hydride Complexes Supported by the Diarylamido/Bis(phosphine) PNP Ligand and Attempts at Using (PNP)Ru and (PNP)Os Complexes in C-H Borylation Catalysis

Patricio Castillo, Bryan J. Foley, Samuel R. Lee, Billy J. McCulloch, Nattamai Bhuvanesh, and Oleg V. Ozerov*

Department of Chemistry, Texas A&M University, College Station, Texas 77842

ozerov@chem.tamu.edu

Table of Contents

I. X-ray Structural Determination Details. 3
II. NMR Spectra 6
III. SI References 30

I. X-ray Structural Determination Details.

X-Ray data collection, solution, and refinement for 2-Os (CCDC: 2030947).

A Leica MZ 7_5 microscope was used to identify a suitable green block with very well-defined faces with dimensions (max, intermediate, and min) 0.497 x 0.472 x 0.341 mm^3 from a representative sample of crystals of the same habit. The crystal mounted on a nylon loop was then placed in a cold nitrogen stream (Oxford) maintained at 110 K.

A BRUKER APEX 3 X-ray (three-circle) diffractometer was employed for crystal screening, unit cell determination, and data collection. The goniometer was controlled using the APEX3 software suite, v2017.3-0.[1] The sample was optically centered with the aid of a video camera such that no translations were observed as the crystal was rotated through all positions. The detector was set at 6.0 cm from the crystal sample (APEX2, 512x512 pixel). The X-ray radiation employed was generated from a Mo sealed X-ray tube (K_α = 0.71073Å with a potential of 40 kV and a current of 40 mA).

Sixty data frames were taken at widths of 1.0°. These reflections were used in the auto-indexing procedure to determine the unit cell. A suitable cell was found and refined by nonlinear least squares and Bravais lattice procedures. The unit cell was verified by examination of the *h k l* overlays on several frames of data. No super-cell or erroneous reflections were observed.

After careful examination of the unit cell, an extended data collection procedure (4 sets) was initiated using omega scans.

Integrated intensity information for each reflection was obtained by reduction of the data frames with the program APEX3.[1] The integration method employed a three-dimensional profiling algorithm and all data were corrected for Lorentz and polarization factors, as well as for crystal

decay effects. Finally, the data was merged and scaled to produce a suitable data set. The absorption correction program SADABS[2] was employed to correct the data for absorption effects. Systematic reflection conditions and statistical tests of the data suggested the space group *P*-1. A solution was obtained readily (Z=1; Z'=0.5) using XT/XS in APEX3.[1,3] Hydrogen atoms were placed in idealized positions and were set riding on the respective parent atoms. All non-hydrogen atoms were refined with anisotropic thermal parameters. Absence of additional symmetry and voids were confirmed using PLATON (ADDSYM).[4] The structure was refined (weighted least squares refinement on F^2) to convergence.[3,5]

X-Ray data collection, solution, and refinement for 5-Os (CCDC: 2030948).

A Leica MZ 7_5 microscope was used to identify a suitable colorless block with very well defined faces with dimensions (max, intermediate, and min) 0.194 x 0.152 x 0.143 mm^3 from a representative sample of crystals of the same habit. The crystal mounted on a nylon loop was then placed in a cold nitrogen stream (Oxford) maintained at 100 K.

A BRUKER Quest X-ray (fixed-Chi geometry) diffractometer was employed for crystal screening, unit cell determination, and data collection. The goniometer was controlled using the APEX3 software suite.[1] The sample was optically centered with the aid of a video camera such that no translations were observed as the crystal was rotated through all positions. The X-ray radiation employed was generated from a Mo-Iμs X-ray tube (K_α = 0.71073Å).

45 data frames were taken at widths of 1°. These reflections were used to determine the unit cell. The unit cell was verified by examination of the *h k l* overlays on several frames of data. No super-cell or erroneous reflections were observed.

After careful examination of the unit cell, an extended data collection procedure (4 sets) was initiated using omega scans.

Integrated intensity information for each reflection was obtained by reduction of the data frames with the program APEX3.[1] The integration method employed a three dimensional profiling algorithm and all data were corrected for Lorentz and polarization factors, as well as for crystal decay effects. Finally, the data was merged and scaled to produce a suitable data set. The absorption correction program SADABS[2] was employed to correct the data for absorption effects.

Systematic reflection conditions and statistical tests of the data suggested the space group *P*-1. A solution was obtained readily (Z=4; Z'=2) using XT/XS in APEX3.[1,3] Partially occupied solvent, pentane, was found. Hydrogen atoms were placed in idealized positions and were set riding on the respective parent atoms. All non-hydrogen atoms were refined with anisotropic thermal parameters. Elongated thermal ellipsoids on the solvent and the residual electron densities suggested disorder, which was modeled successfully between two positions. Initially the occupancies of the solvent at both the positions were refined independently and were then fixed at the refined values during the final least squares refinement cycles. Absence of additional symmetry were confirmed using PLATON (ADDSYM).[4] The structure was refined (weighted least squares refinement on F^2) to convergence.[3,4] CHECKCIF suggested solvent accessible voids. However, our efforts to SQUEEZE the solvent with “solvent mask” in OLEX2 did not improve the results.

II. NMR Spectra

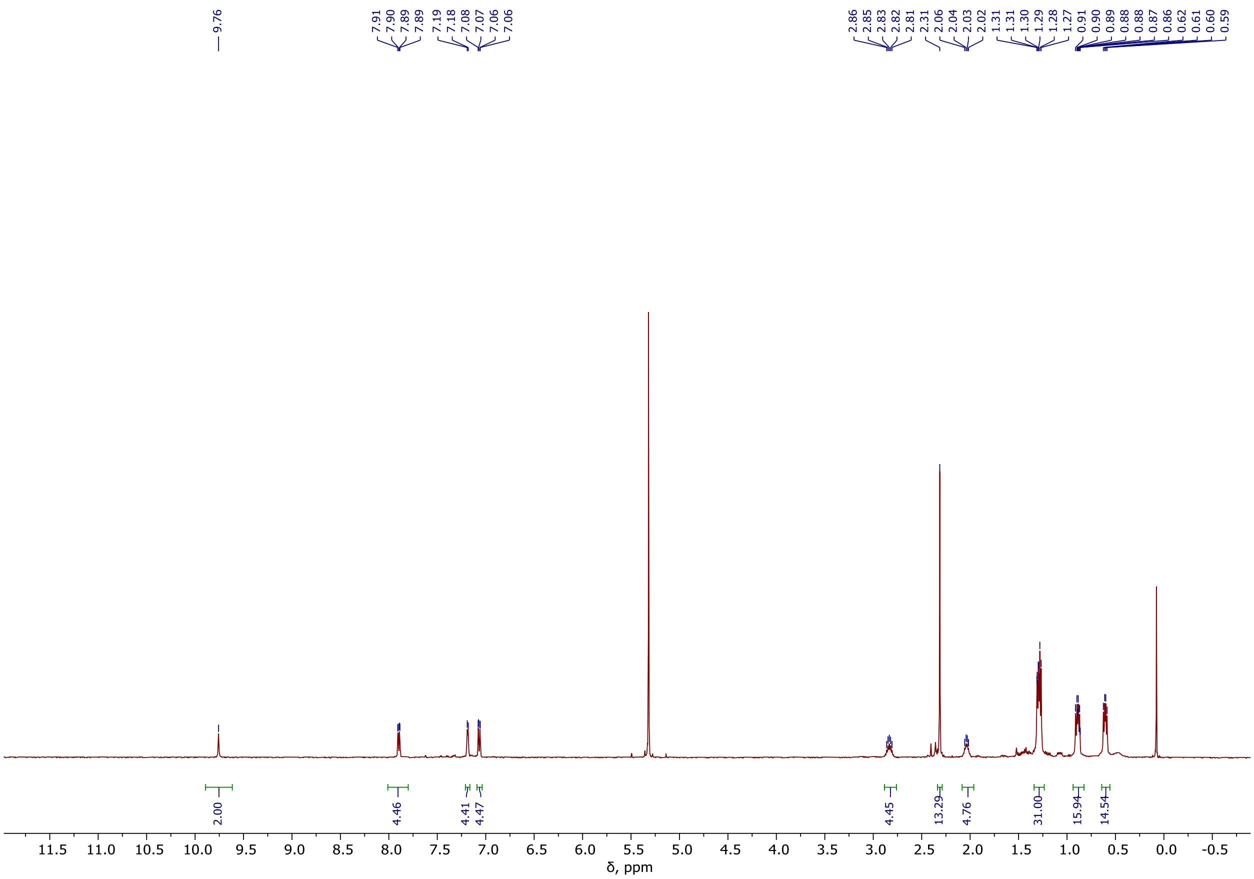

Figure S1. ^{1}H NMR (500 MHz, CD_2Cl_2) spectrum of **2-Os**. Silicone grease present at δ 0.07.

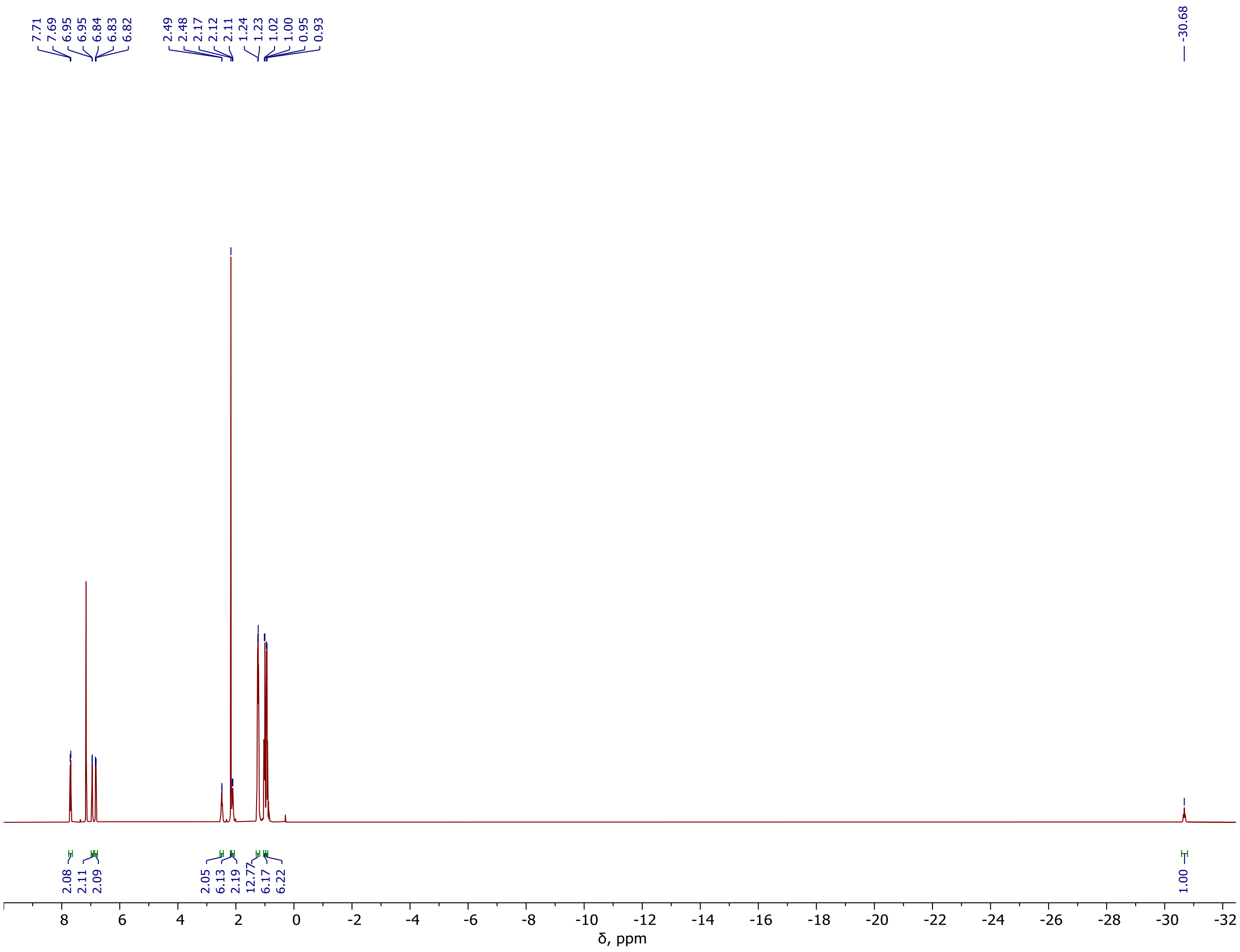

Figure S2. ^{1}H NMR (400 MHz, C_6D_6) spectrum of **3-Os**.

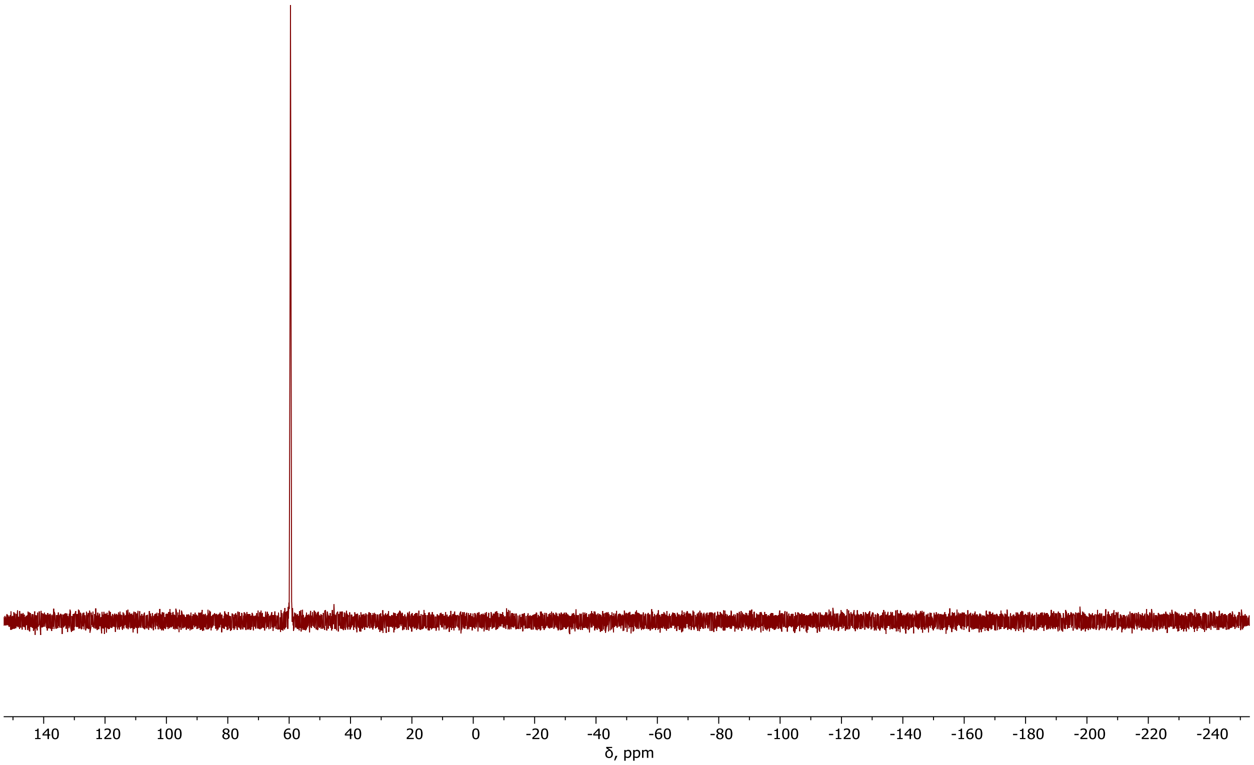

Figure S3. $^{31}P\{^{1}H\}$ NMR (162 MHz, C_6D_6) spectrum of **3-Os**.

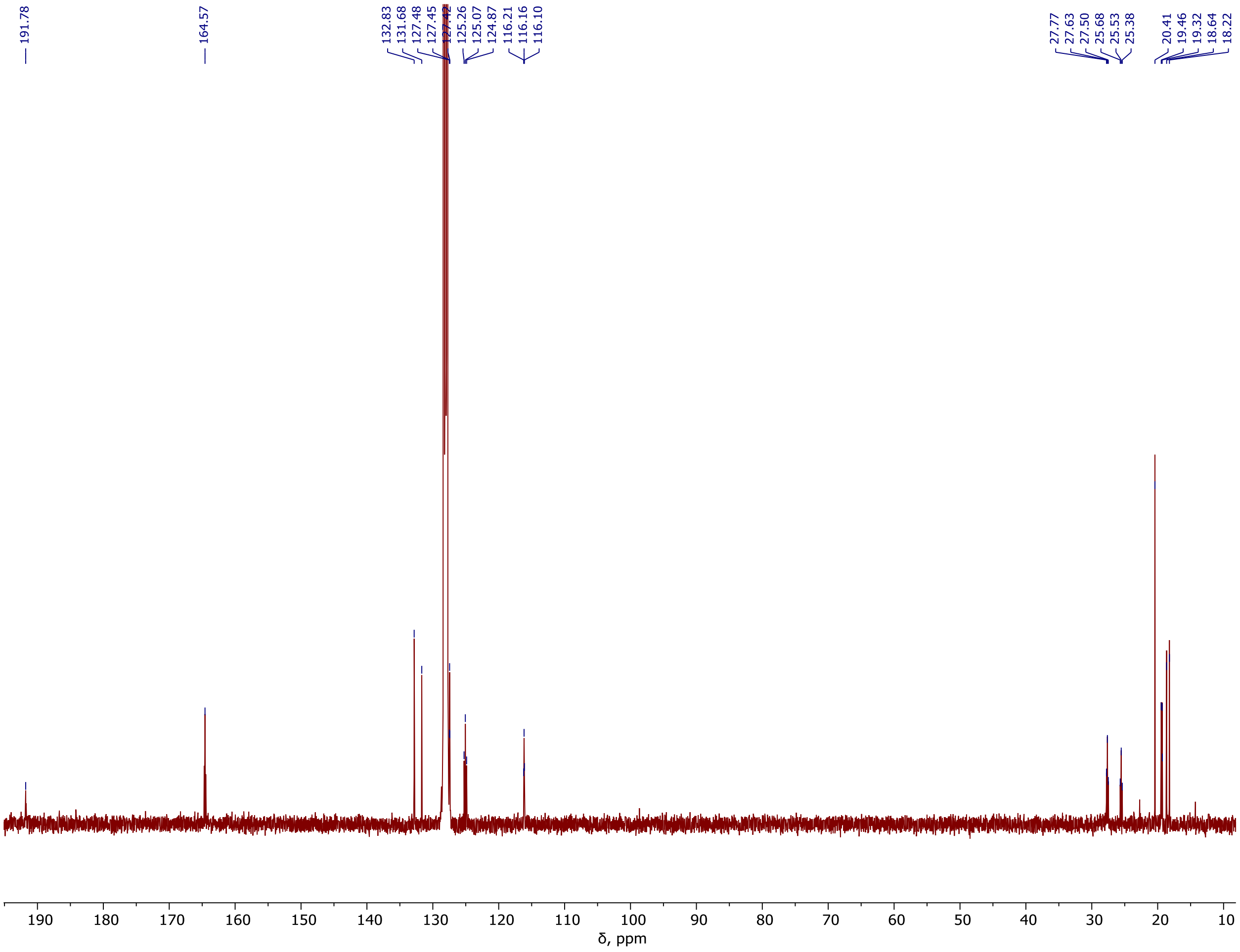

Figure S4. $^{13}C\{^1H\}$ NMR (101 MHz, C_6D_6) spectrum of **3-Os**.

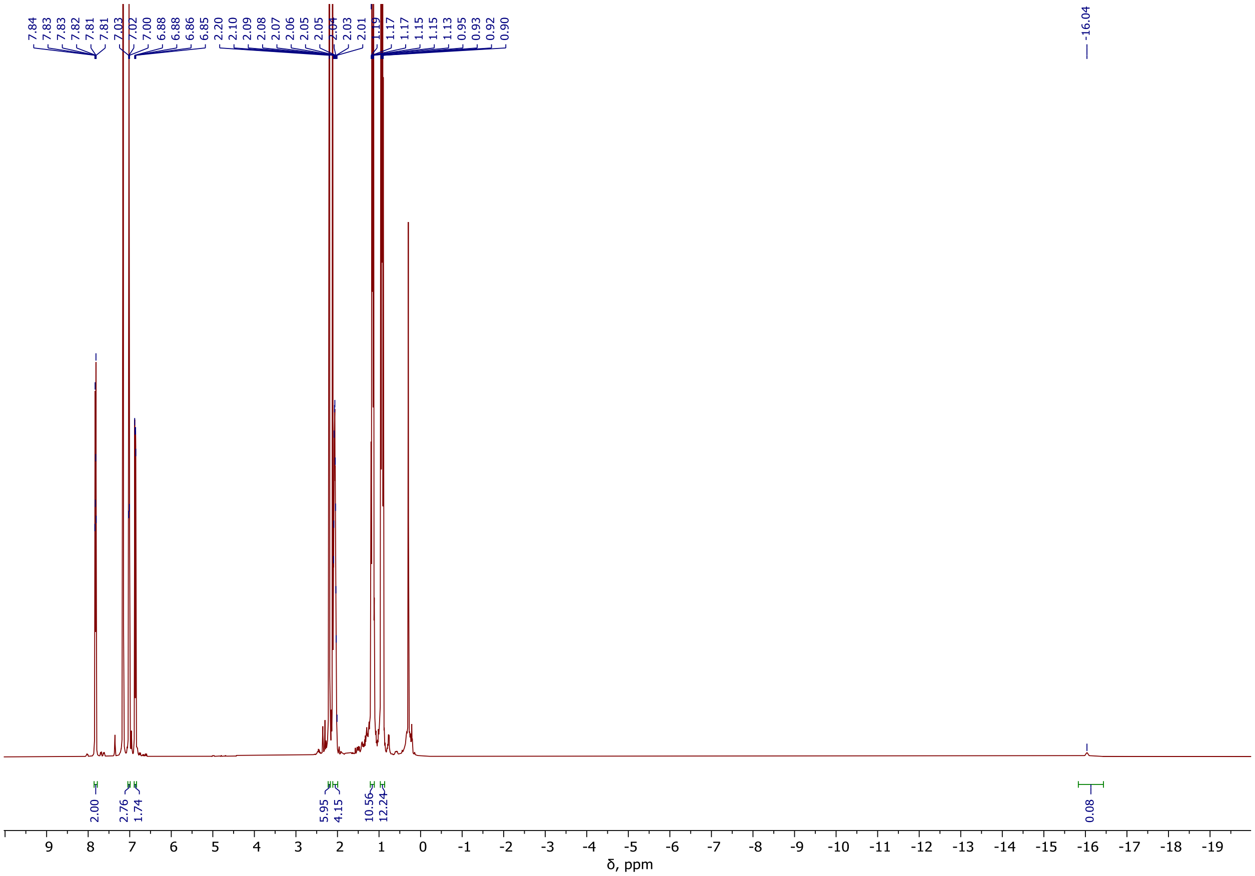

Figure S5. ^{1}H NMR (400 MHz, C_6D_6) spectrum of **4-Os**.

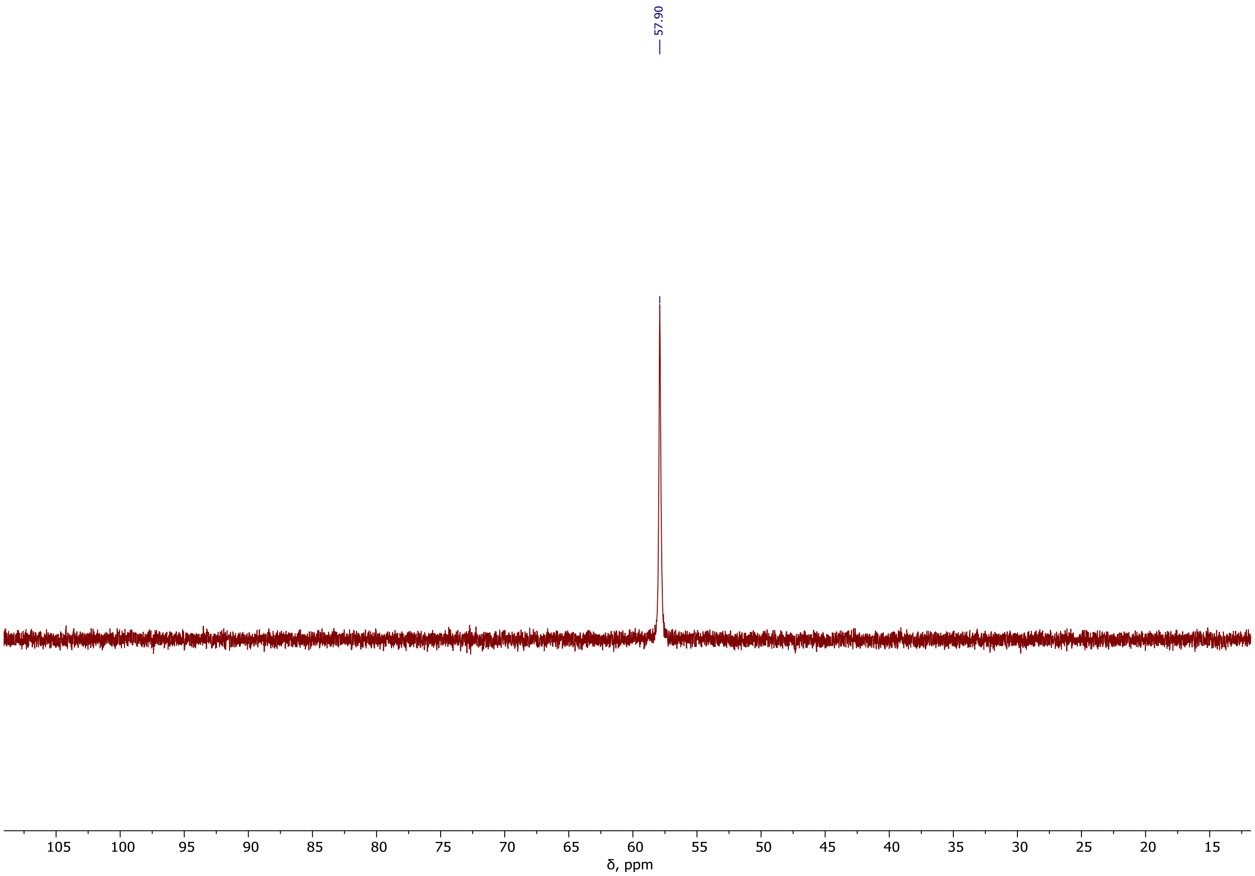

Figure S6. $^{31}P\{^{1}H\}$ NMR (121 MHz, C_6D_6) spectrum of **4-Os**.

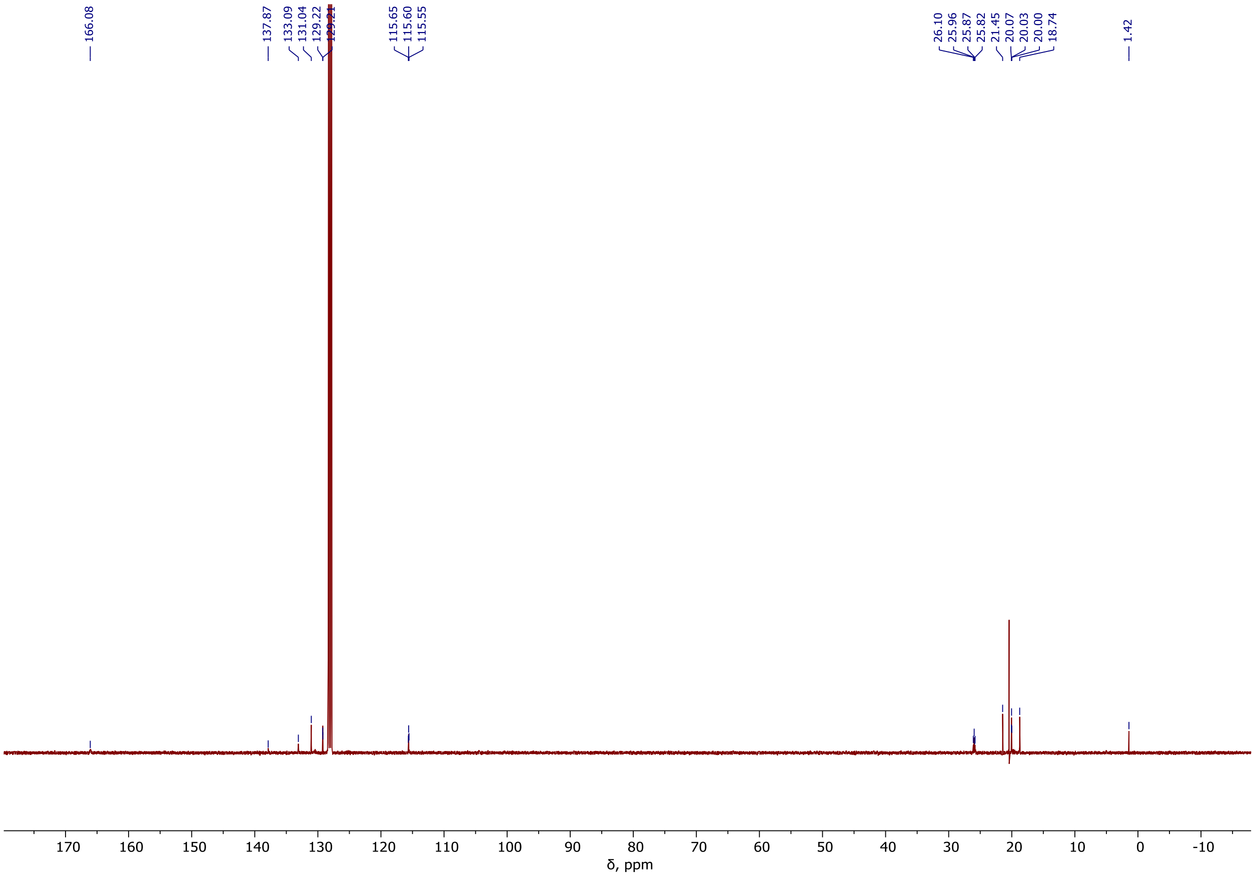

Figure S7. $^{13}C\{^{1}H\}$ NMR (101 MHz, C_6D_6) spectrum of **4-Os**.

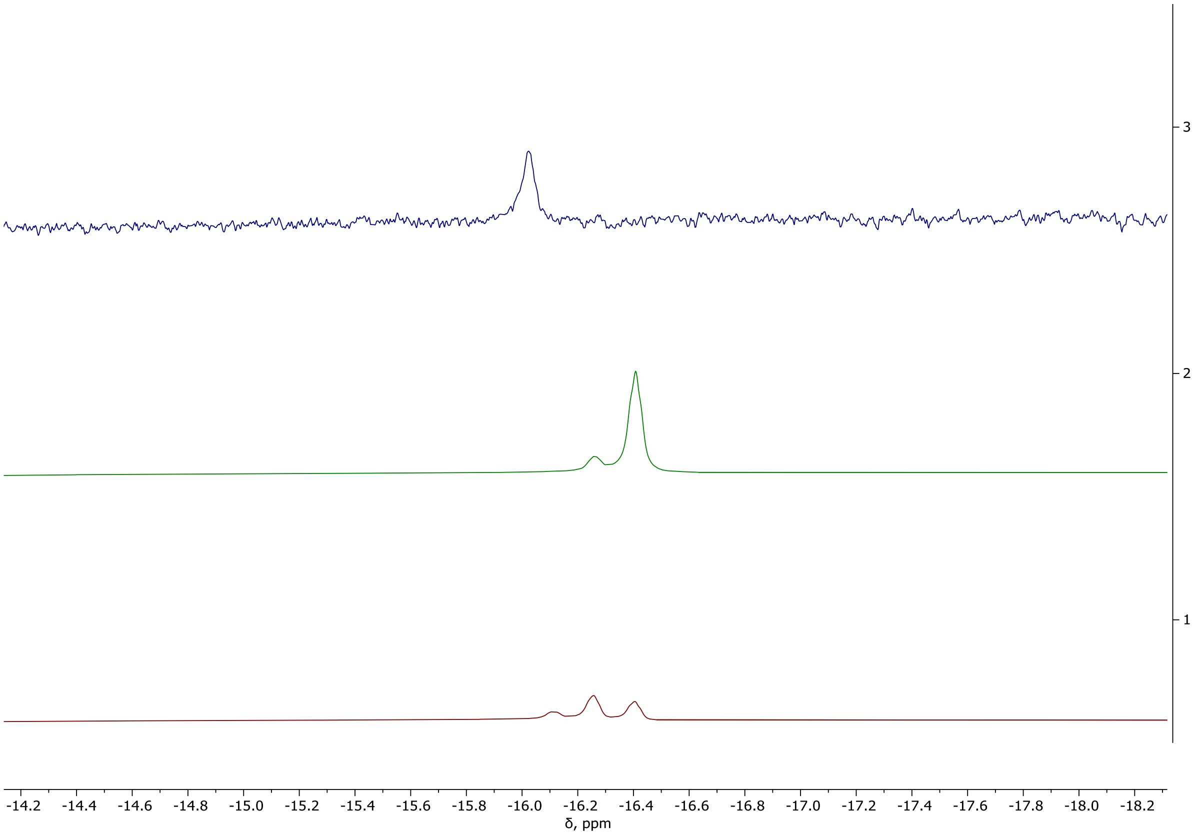

Figure S8. ^{1}H NMR (500 MHz, C_6D_6) spectra of observed isotopomers of **4-Os** in C_6D_6 after 20 minutes (top), and 70 minutes after addition of C_6H_5F (middle), and 20 hours after addition of C_6H_5F (bottom). Area shown is the hydride region.

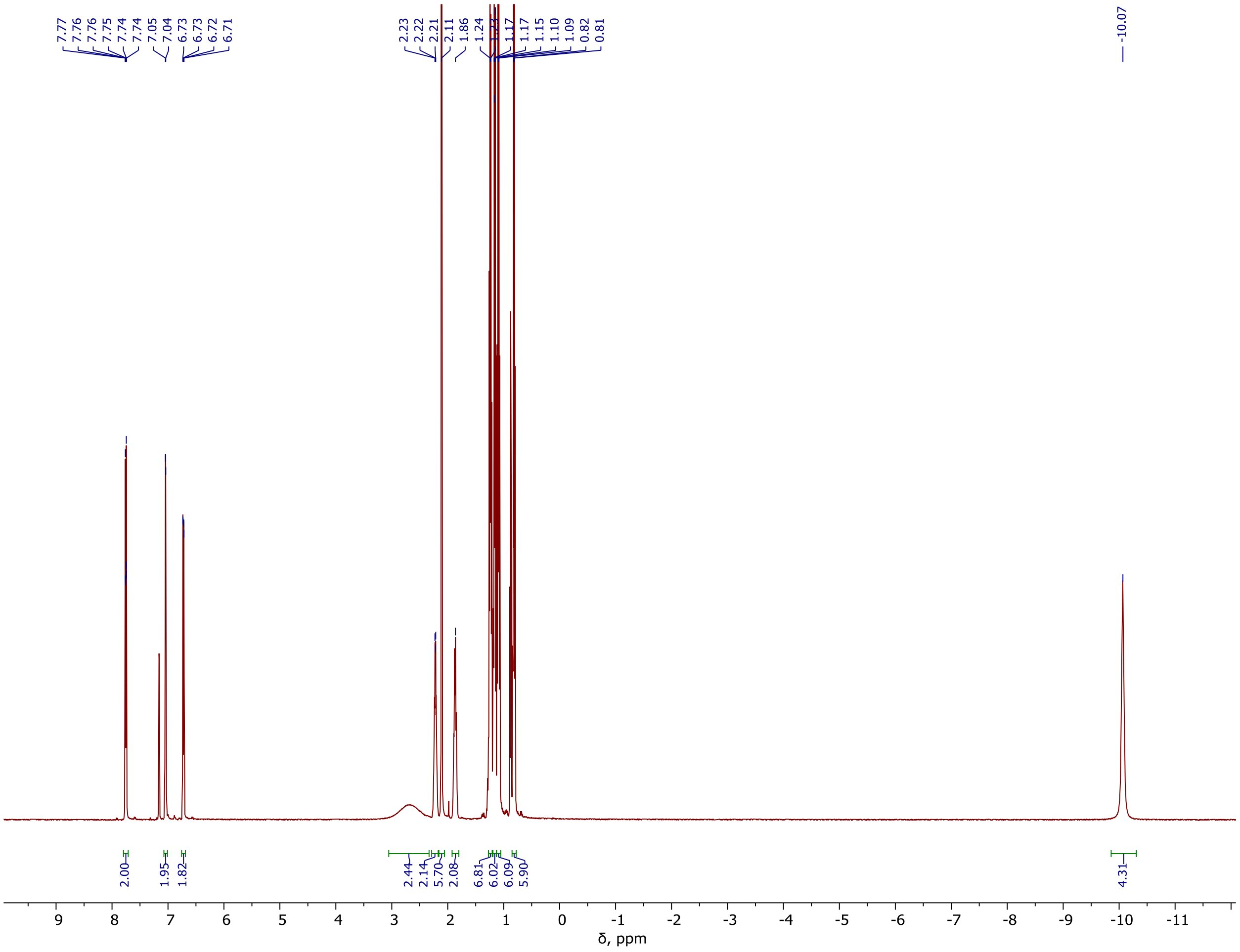

Figure S9. ^{1}H NMR (500 MHz, C_6D_6) spectrum of **5-Os**.

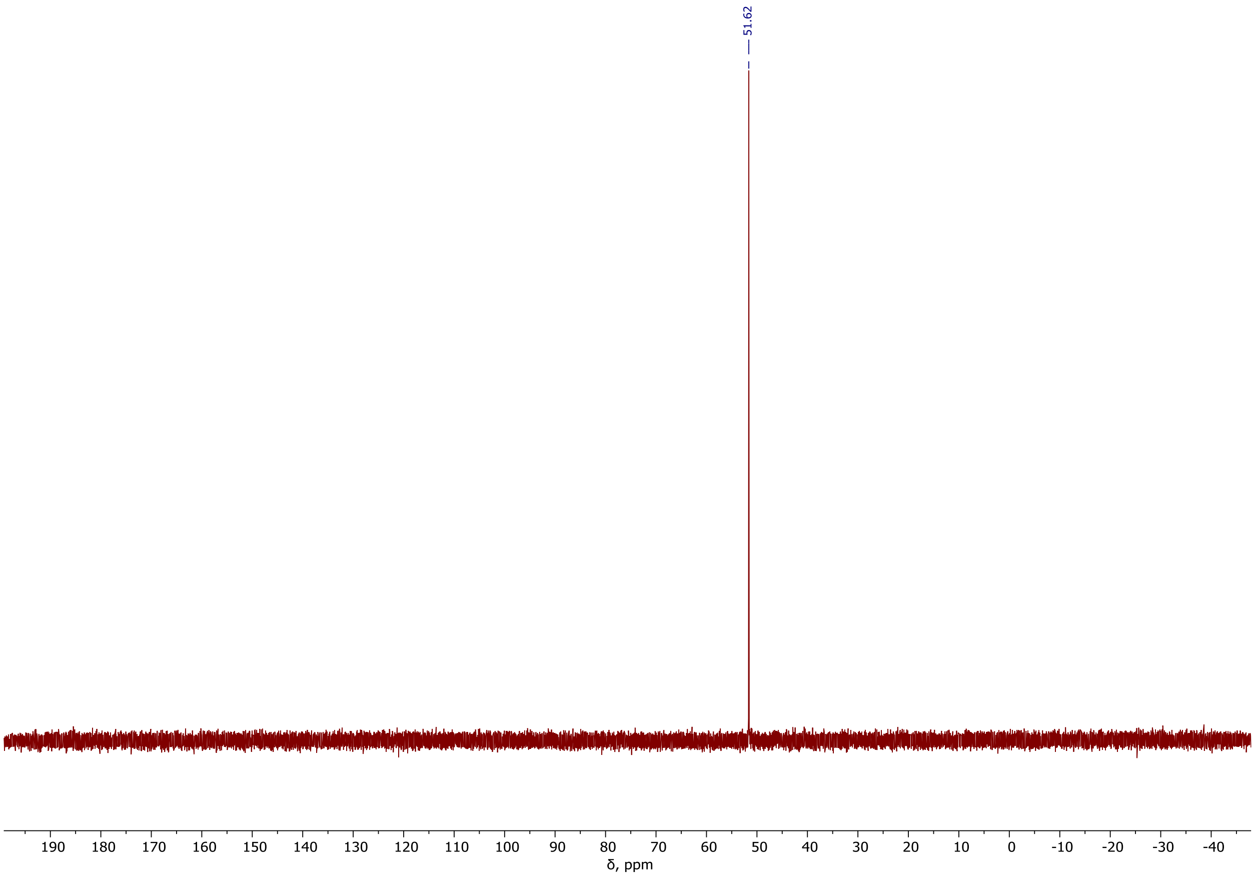

Figure S10. $^{31}P\{^{1}H\}$ NMR (202 MHz, C_6D_6) spectrum of **5-Os.**

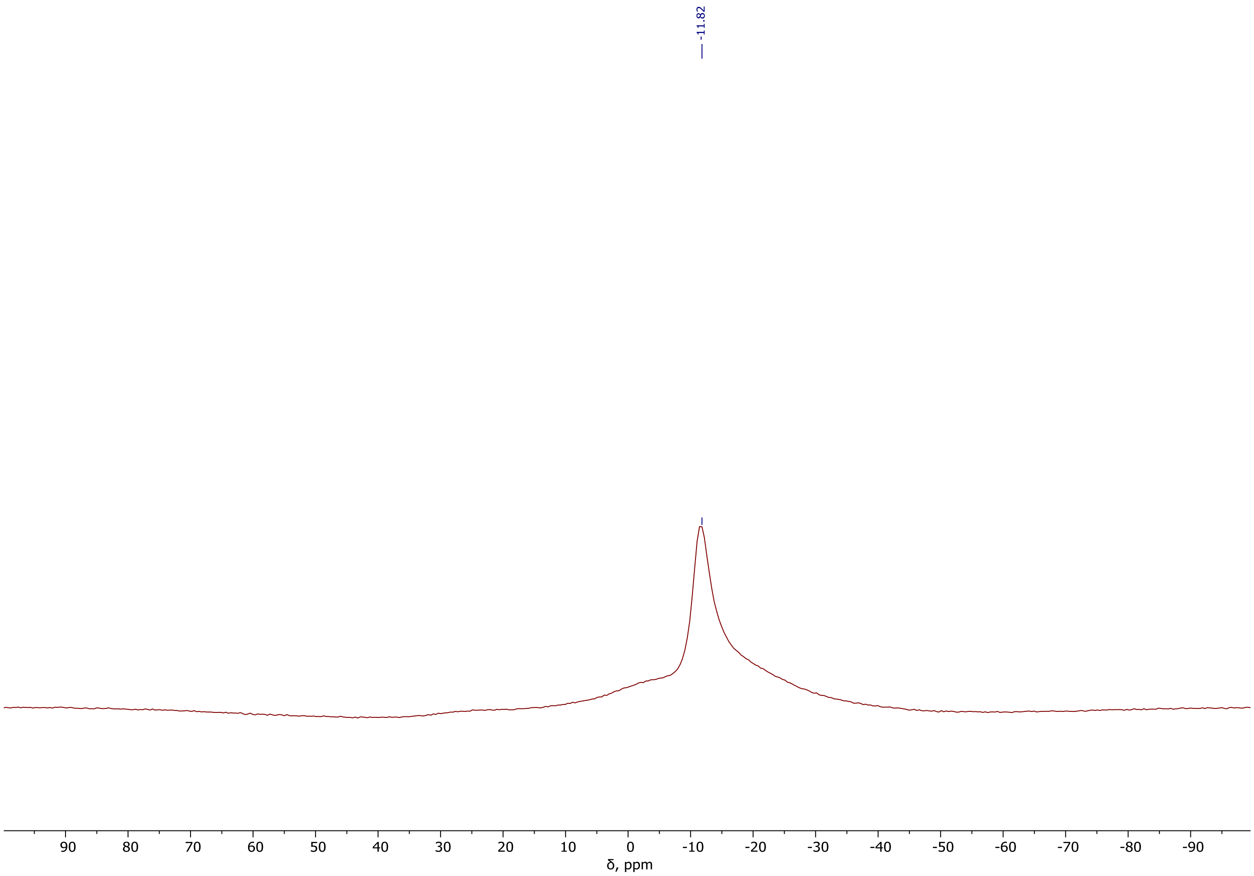

Figure S11. ^{11}B NMR (128 MHz, C_6D_6) spectrum of **5-Os.**

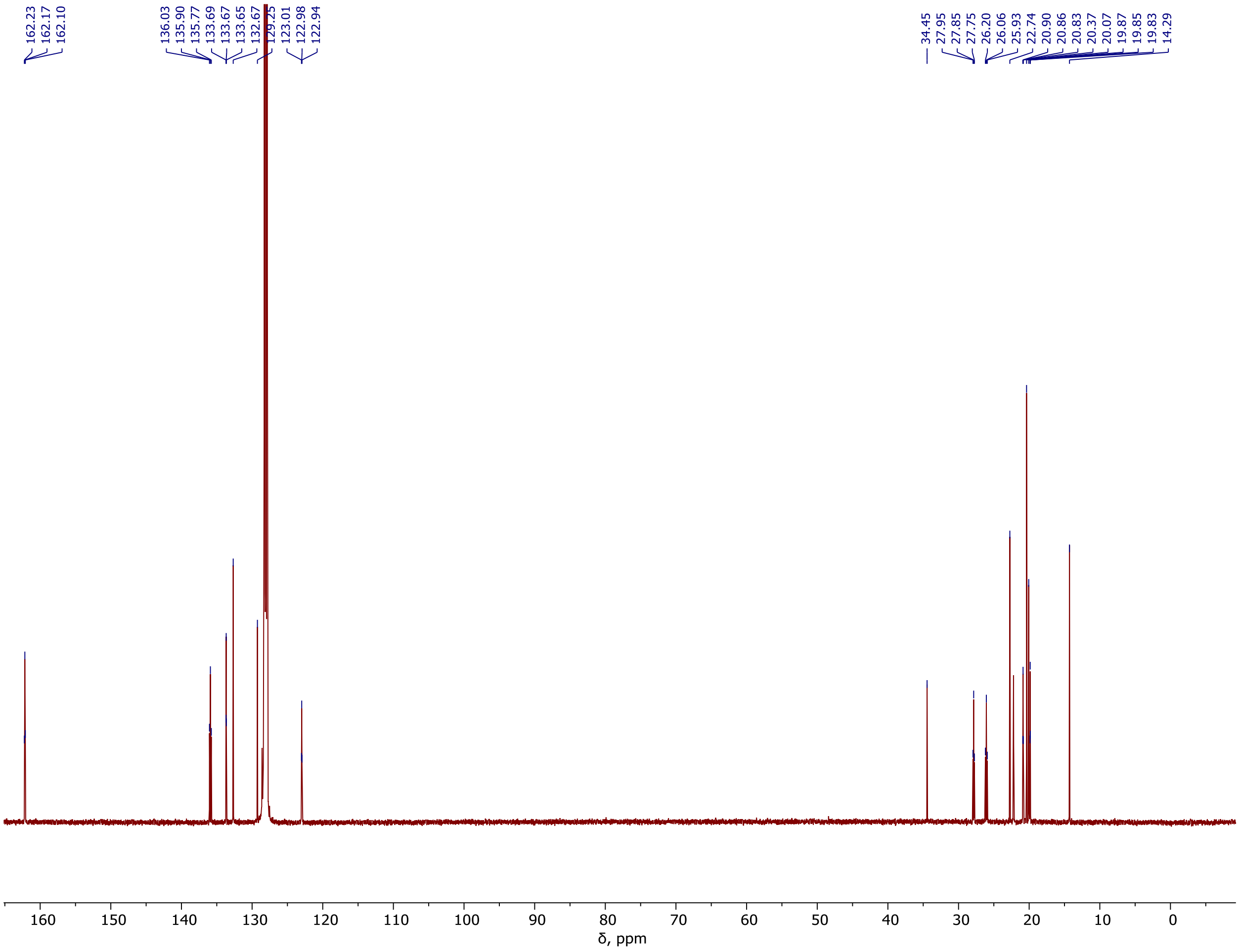

Figure S12. $^{13}C\{^{1}H\}$ NMR (126 MHz, C_6D_6) spectrum of **5-Os**.

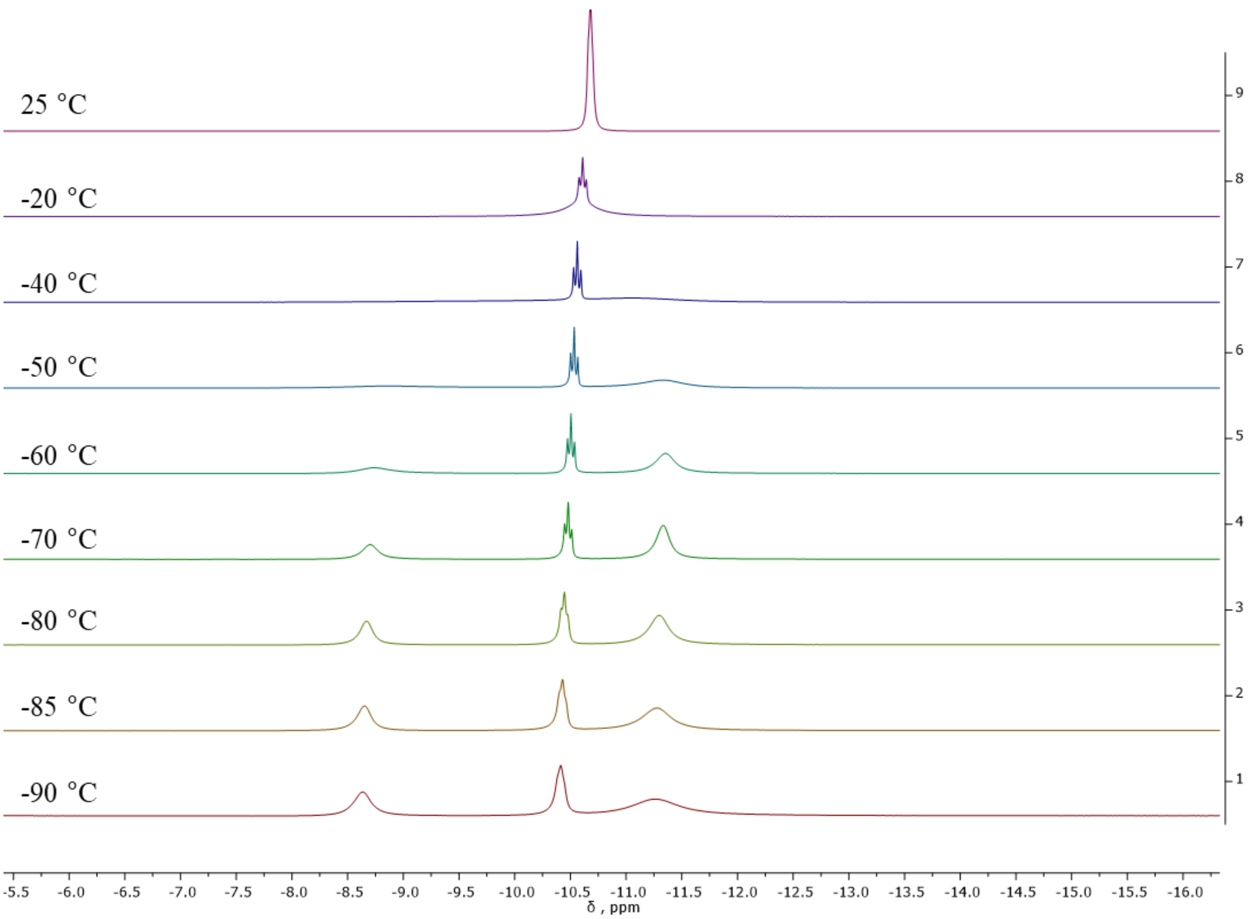

Figure S13. Variable temperature ^{1}H NMR (500 MHz, toluene-d_8) of **5-Os** from 25 °C to -90 °C. Area shown is the hydride region.

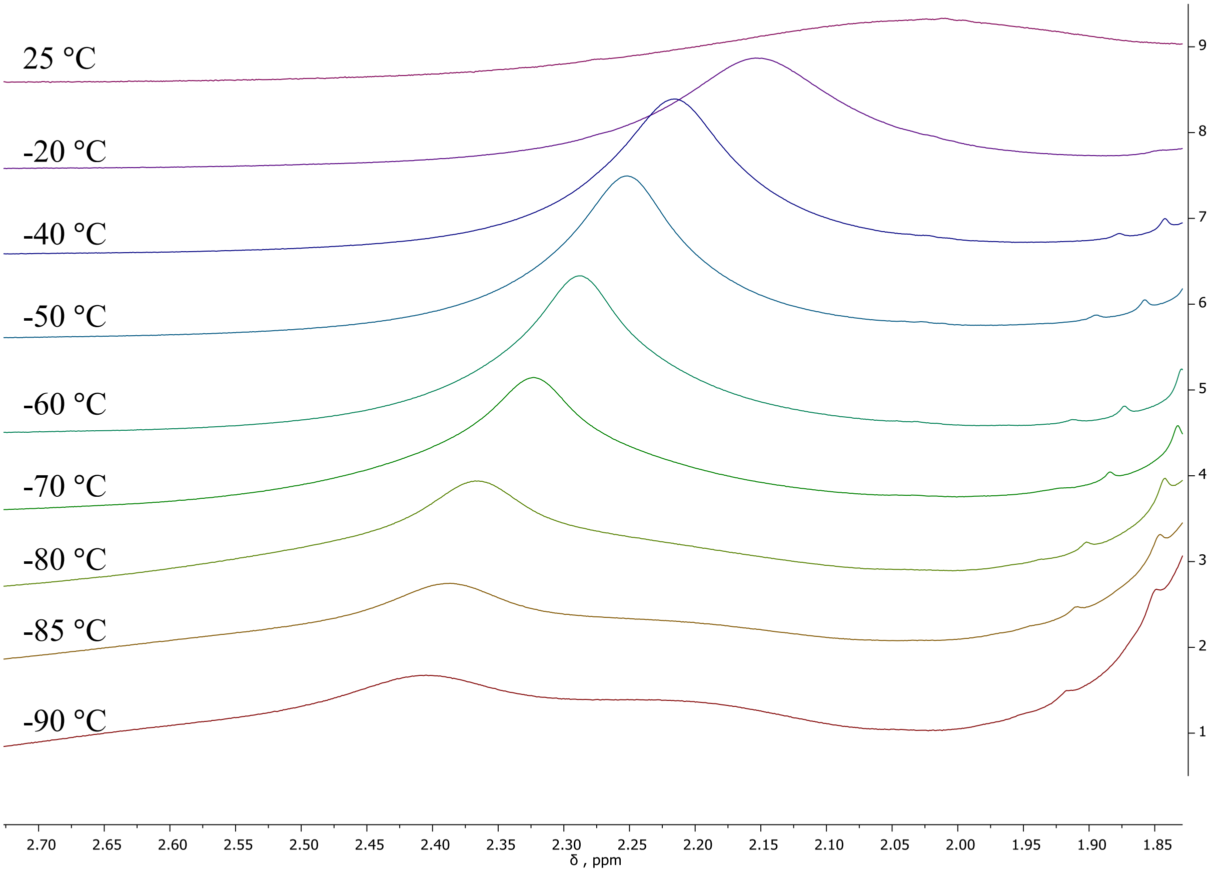

Figure S14. Variable temperature ^{1}H NMR (500 MHz, toluene-d_8) of **5-Os** from 25 °C to -90 °C. Area shown is B-H signal.

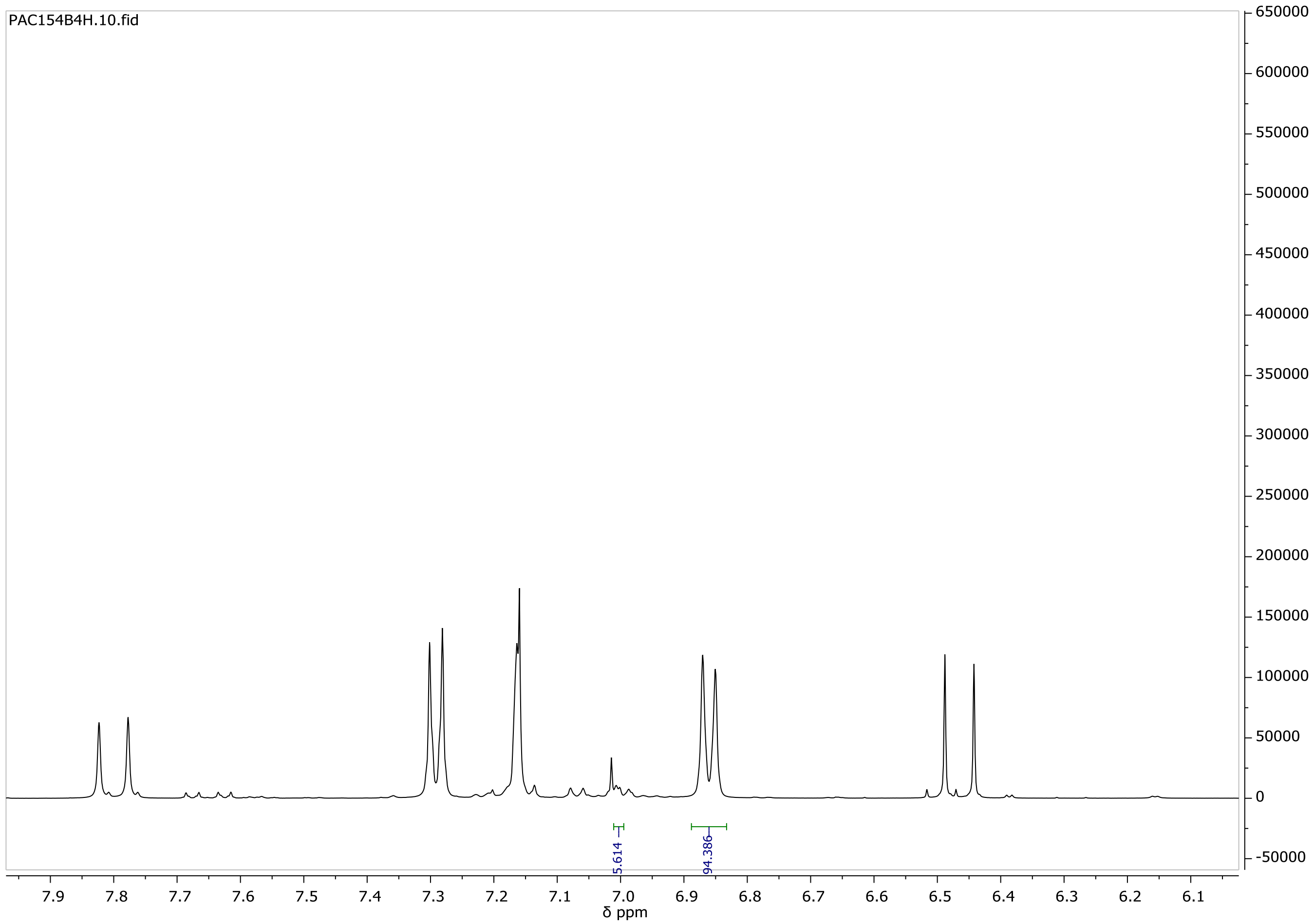

Figure S15. ^{1}H NMR (400 MHz, C_6D_6) spectrum of two alkenylboronate products resulting from the reaction of 4-ethynyltoluene and HBpin (1 mol % **3-Ru**).

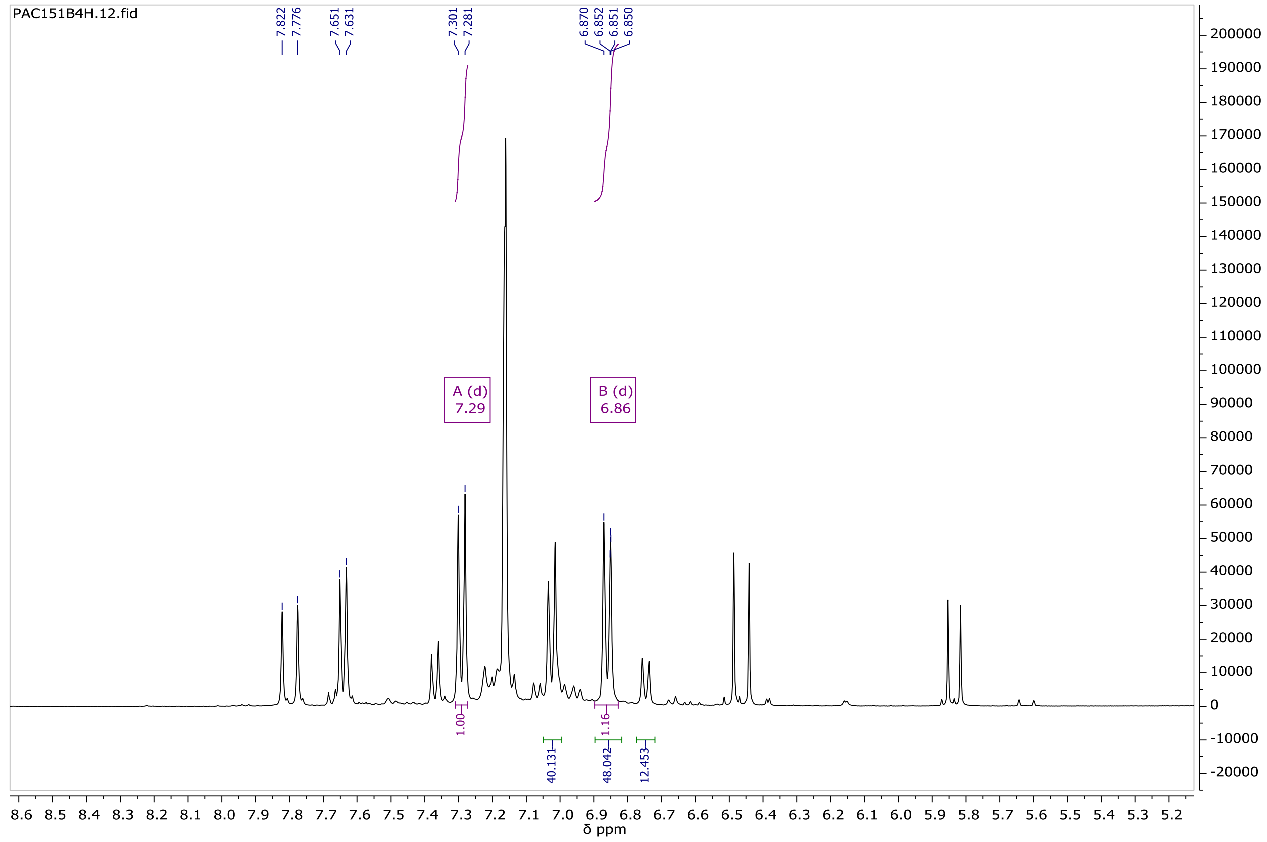

Figure S16. ^{1}H NMR (400 MHz, C_6D_6) spectrum of two alkenylboronate products resulting from the reaction of 4-ethynyltoluene and HBpin (1 mol % **4-Ru**).

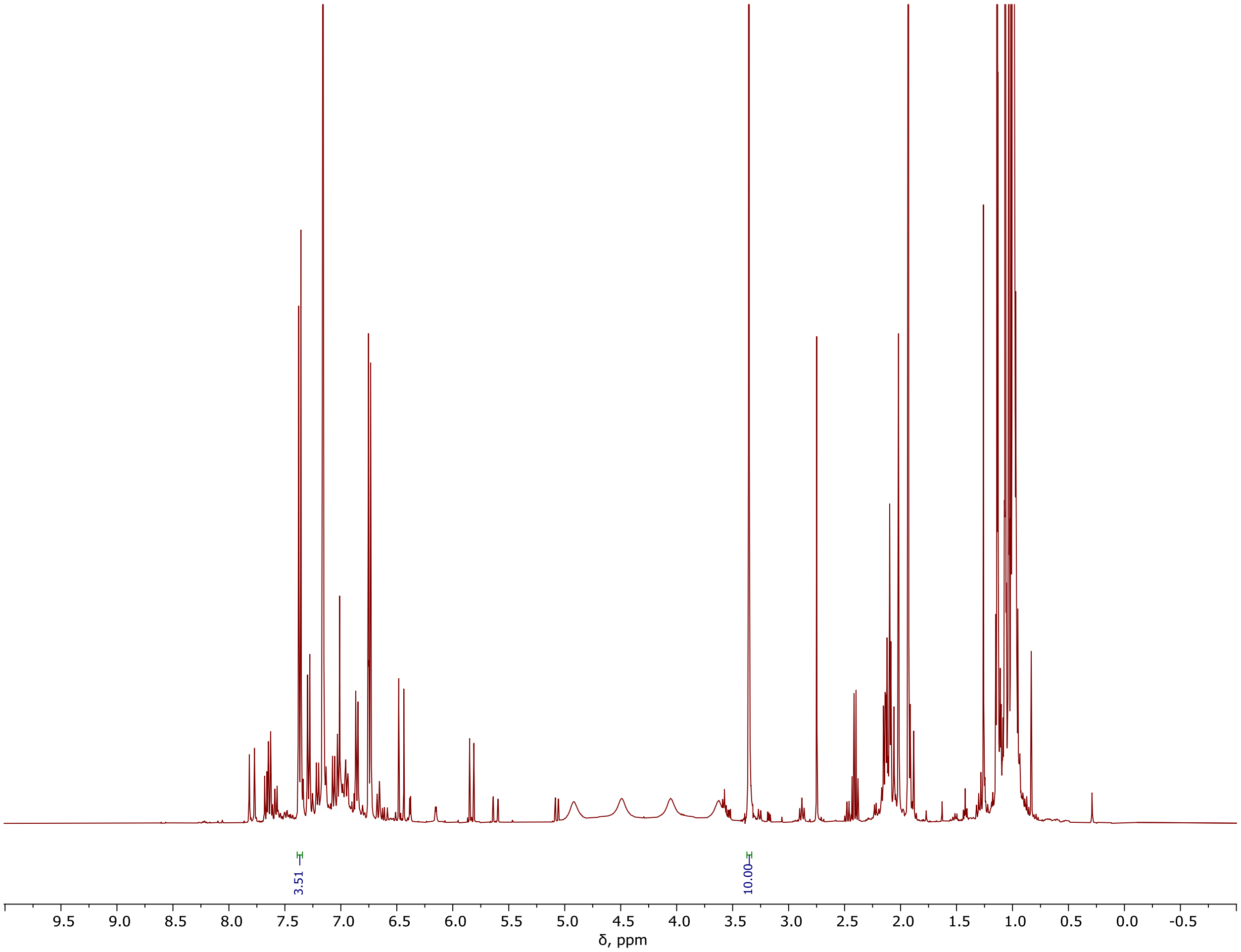

Figure S17. ^{1}H NMR (400 MHz, C_6D_6) spectrum of two alkenylboronate products resulting from the reaction of 4-ethynyltoluene and HBpin (1 mol % **3-Os**).

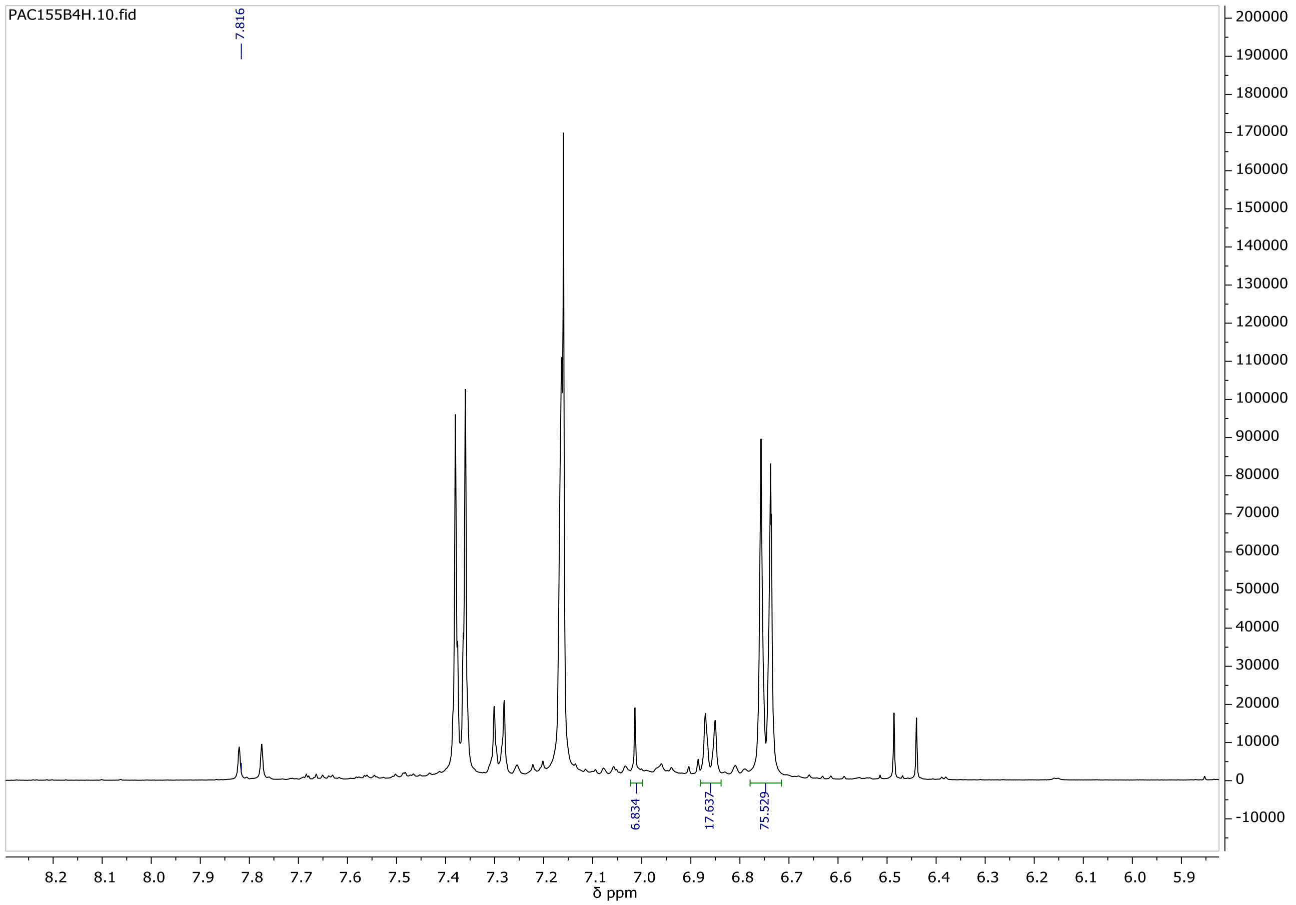

Figure S18. ^{1}H NMR (400 MHz, C_6D_6) spectrum of two alkenylboronate products resulting from the reaction of 4-ethynyltoluene and HBpin (1 mol % **4-Os**).

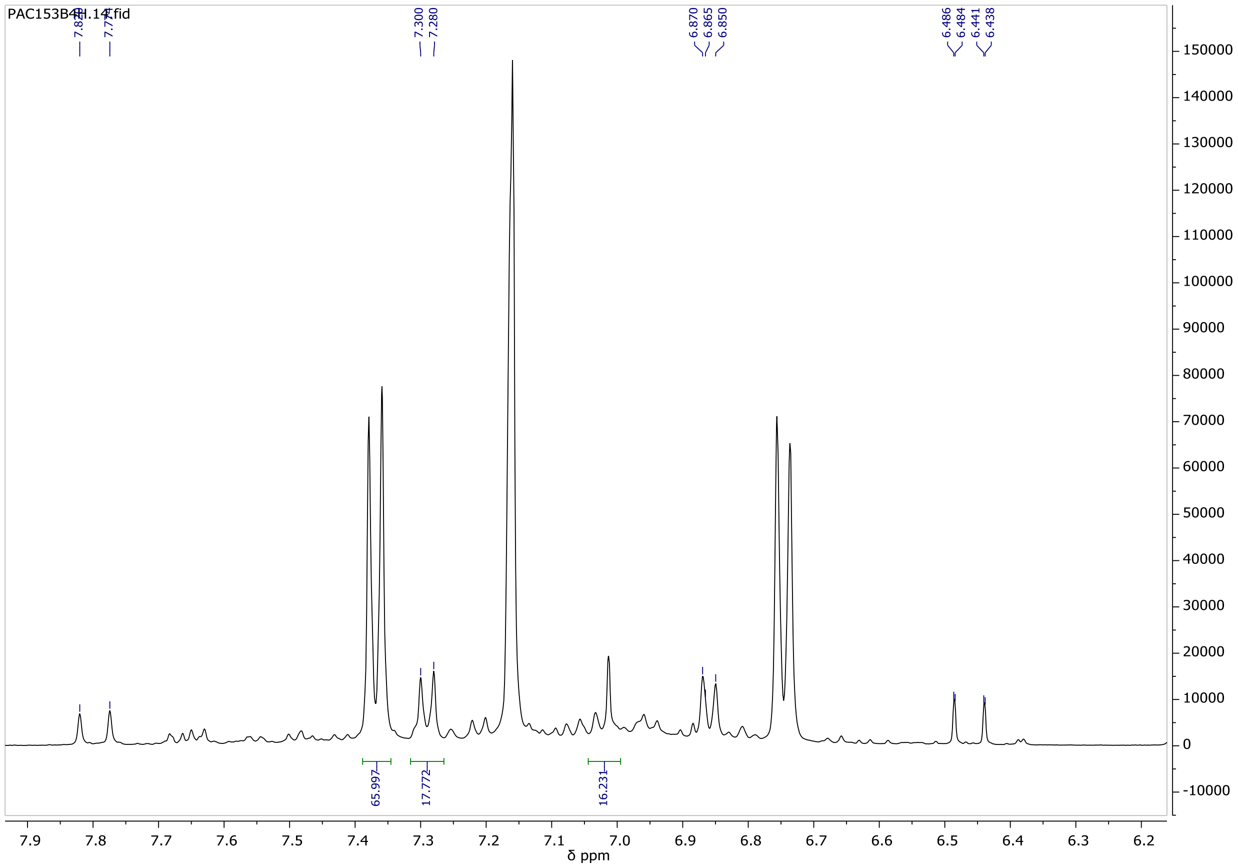

Figure S19. ^{1}H NMR (400 MHz, C_6D_6) spectrum of two alkenylboronate products resulting from the reaction of 4-ethynyltoluene and HBpin (1 mol **% 5-Os**).

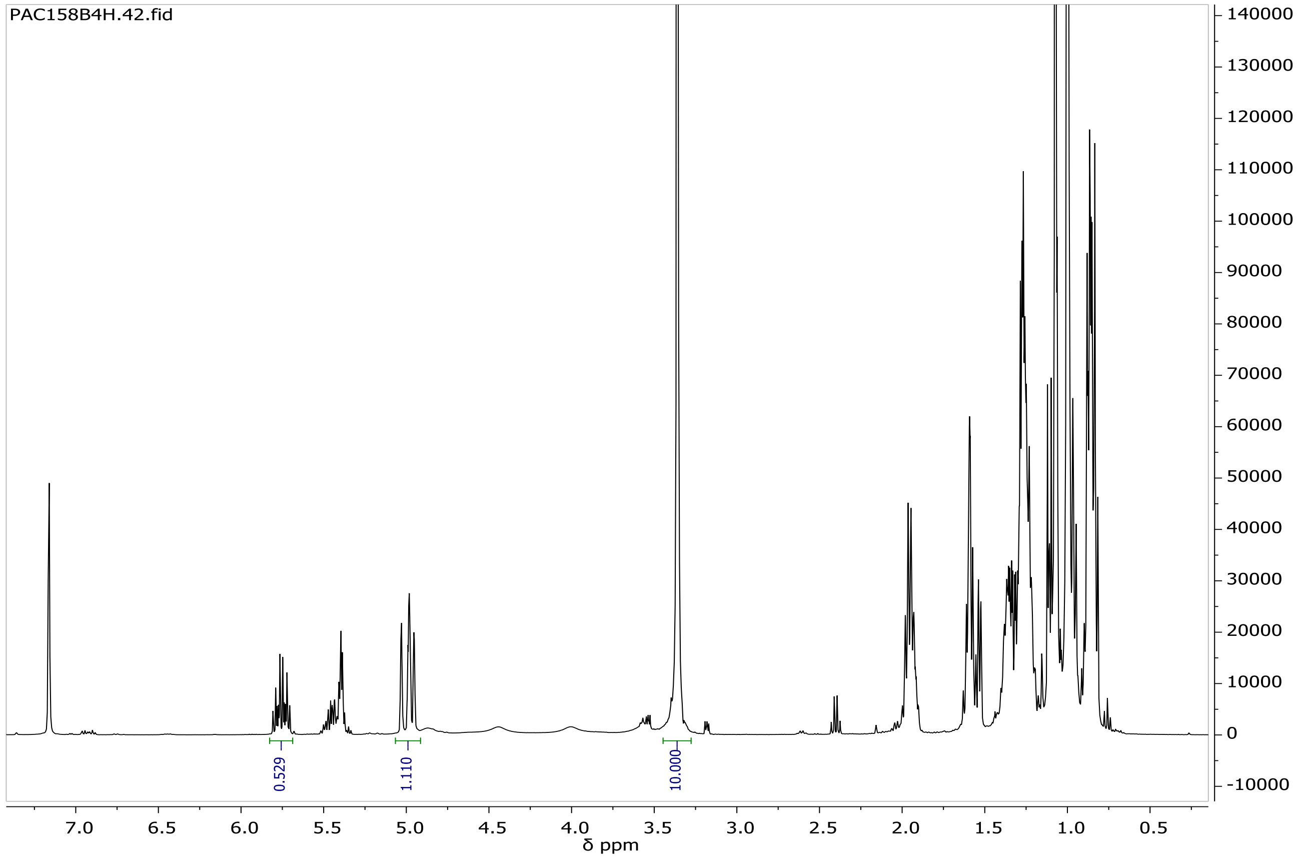

Figure S20. ^{1}H NMR (400 MHz, C_6D_6) spectrum of isomerization of 1-hexene resulting from the reaction of C_6D_6, 1-hexene, and HBpin (1 mol % **3-Ru**).

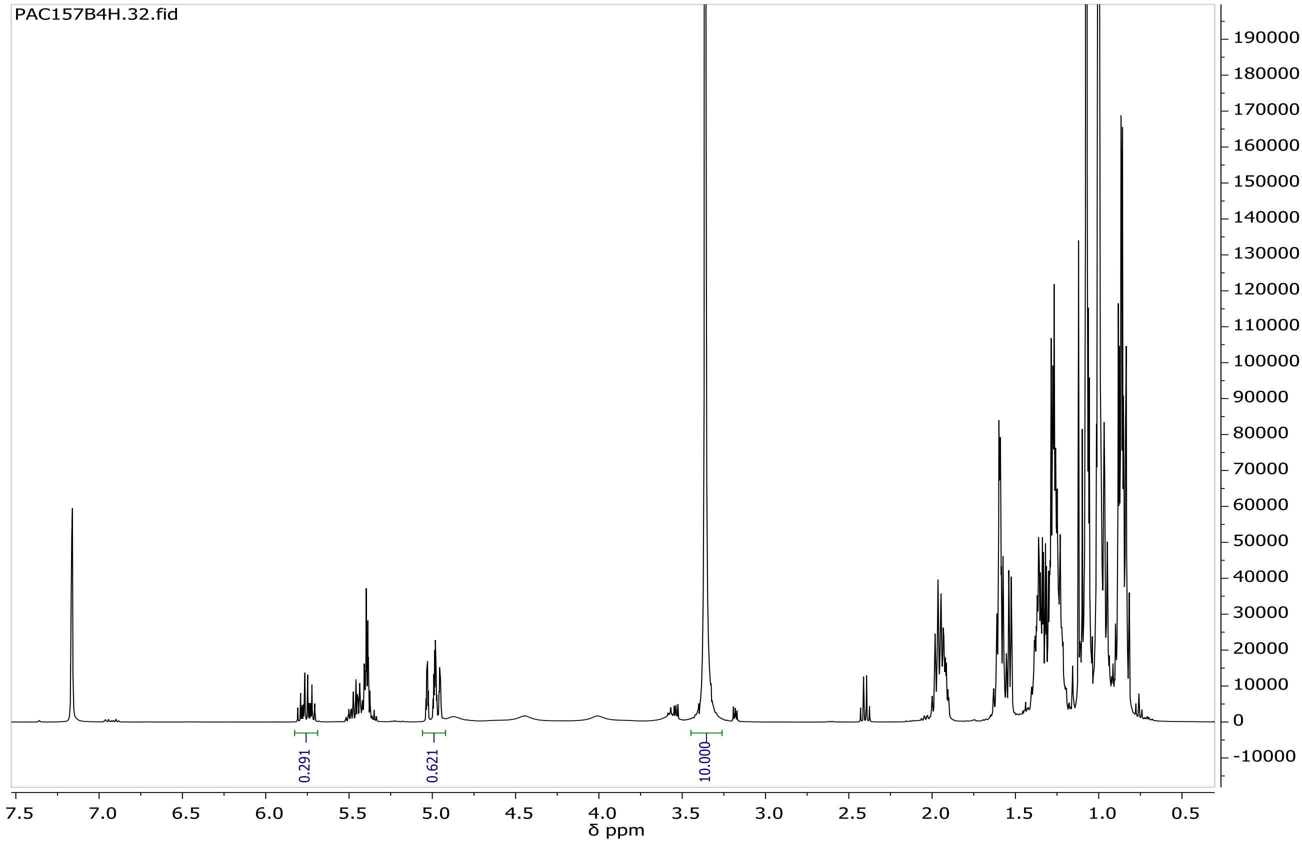

Figure S21. ^{1}H NMR (400 MHz, C_6D_6) spectrum of isomerization of 1-hexene resulting from the reaction of C_6D_6, 1-hexene, and HBpin (1 mol % **4-Ru**).

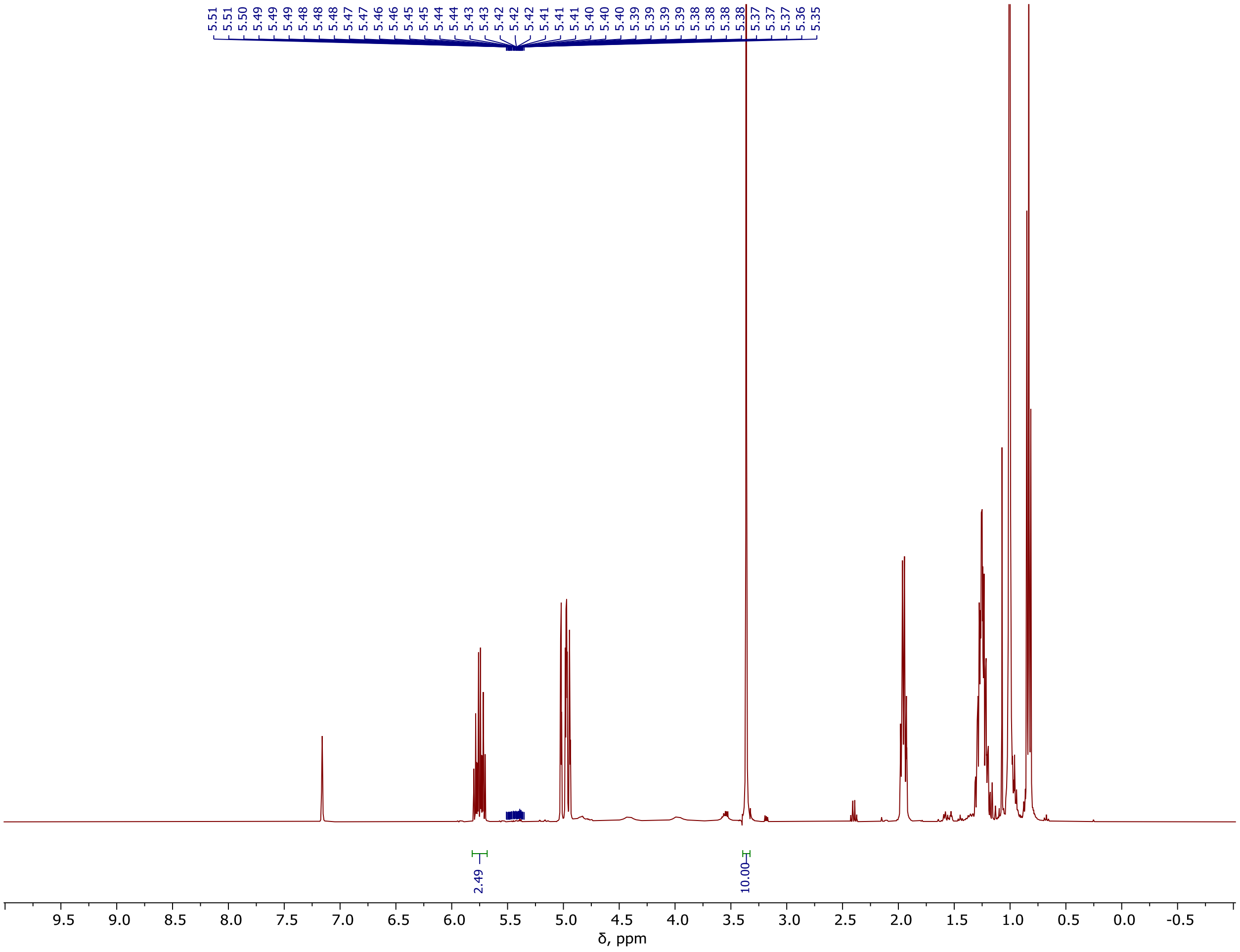

Figure S22. ^{1}H NMR (500 MHz, C_6D_6) spectrum of isomerization of 1-hexene resulting from the reaction of C_6D_6, 1-hexene, and HBpin (1 mol % **3-Os**).

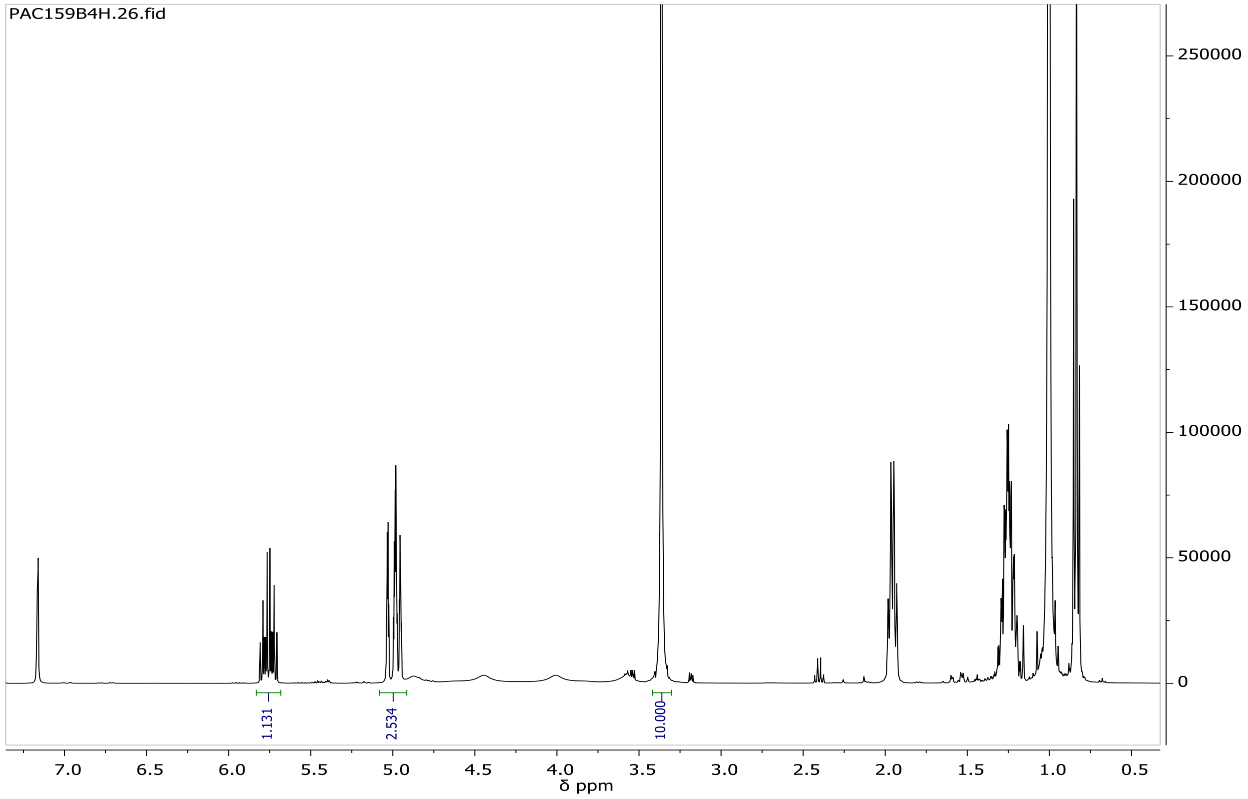

Figure S23. ^{1}H NMR (500 MHz, C_6D_6) spectrum of isomerization of 1-hexene resulting from the reaction of C_6D_6, 1-hexene, and HBpin (1 mol % **4-Os**).

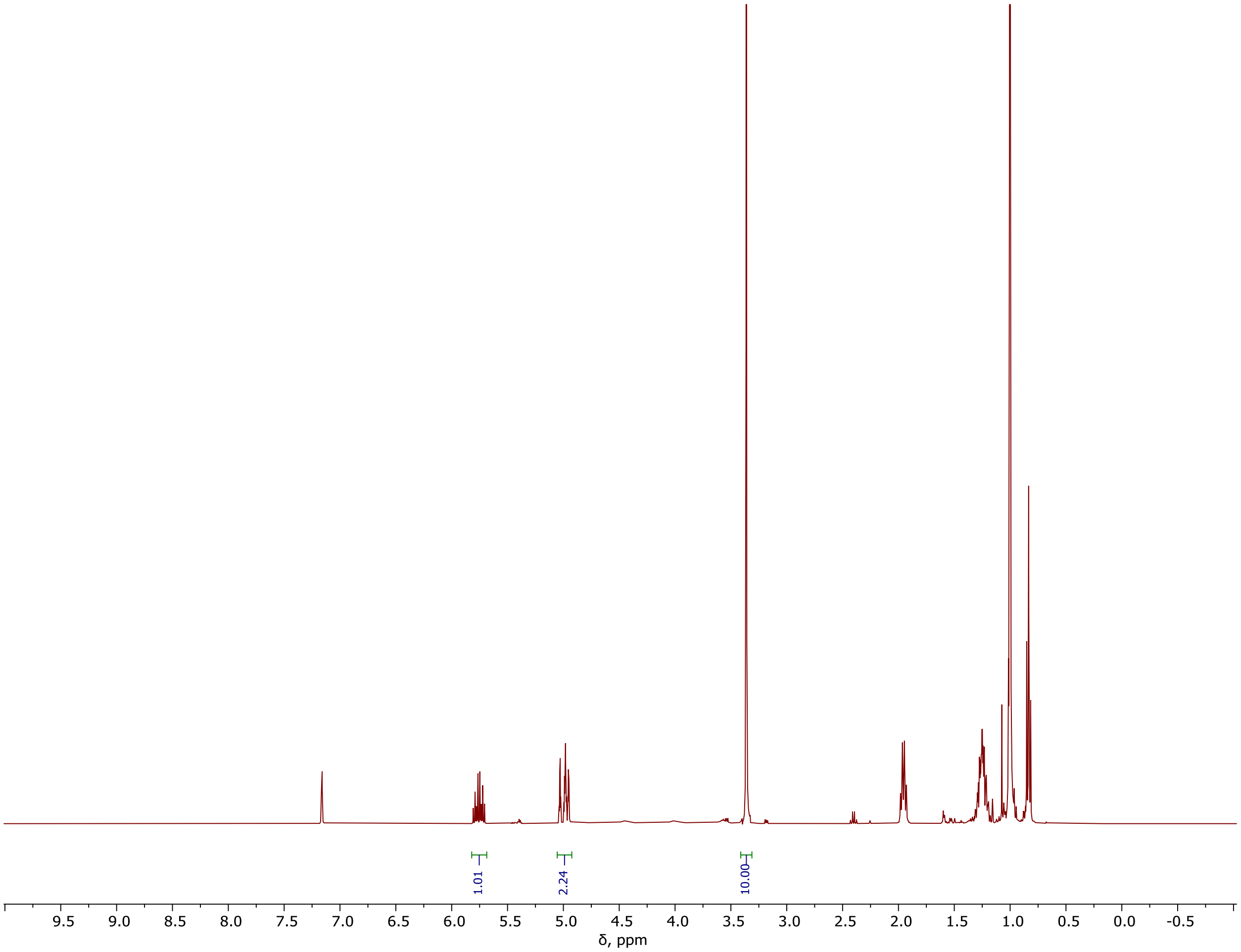

Figure S24. ^{1}H NMR (500 MHz, C_6D_6) spectrum of isomerization of 1-hexene resulting from the reaction of C_6D_6, 1-hexene, and HBpin (1 mol % **5-Os**).

III. SI References

[1] *APEX3: Program for Data Collection on Area Detectors*; Bruker AXS Inc., Madison, WI, USA, 2016.

[2] Sheldrick, G.M. *SADABS: Program for Absorption Correction of Area Detector Frames*; Bruker AXS Inc.: Madison, WI, USA, 2008.

[3] (a) Sheldrick, G. M. A Short History of *SHELX*. *Acta Crystallogr., Sect. A: Found. Crystallogr*. **2008**, *64*, 112−122. (b) Sheldrick, G. M. *SHELXT* – Integrated Space-Group and Crystal Structure Determination. *Acta Crystallogr., Sect. A: Found. Crystallogr.* **2015**, *71*, 3−8. (c) Sheldrick, G. M. Crystal Structure Refinement with *SHELXL*. *Acta Crystallogr., Sect. C: Struct. Chem*. **2015**, *71*, 3−8. (d) *XT, XS*; Bruker AXS Inc.: Madison, WI, USA.

[4] Spek, A. L. Single-Crystal Structure Validation with the Program *PLATON*. *J. Appl. Cryst.* **2003**, *36*, 7-13.

[5] Dolomanov, O. V, Bourhis, L. J., Gildea, R. J., Howard, J. A. K., and Puschmann, H. *OLEX2*: A Complete Structure Solution, Refinement and Analysis Program, *J. Appl. Cryst.* **2009**, *42*, 339-341.
